# Two New Compounds from the Endophytic Fungi of *Dryopteris crassirhizoma* and Their Antimicrobial Activities

**DOI:** 10.3390/molecules28248043

**Published:** 2023-12-12

**Authors:** Ping Hai, Yuan Gao, Lian Yang, Nie Chen, Haiyan Jia, Mengdie Wang, Huan Li, Wenli Jiang, Jian Yang, Rongtao Li

**Affiliations:** 1 Faculty of Life Science and Technology, Kunming University of Science and Technology, Kunming 650500, China; seasea80188532@126.com (P.H.); yl17882559450@126.com (L.Y.); 2 Faculty of Materials and Chemical Engineering, Yibin University, Yibin 644000, China; gymail999@126.com (Y.G.); 17358672819@163.com (N.C.); jhy202303@163.com (H.J.); 15719472645@163.com (M.W.); 15808496152@163.com (H.L.); jwl0306@163.com (W.J.); 3 State Key Laboratory Breeding Base of Dao-di Herbs, National Resource Center for Chinese Materia Medical, China Academy of Chinese Medical Sciences, Beijing 100010, China

**Keywords:** *Dryopteris crassirhizoma*, endophytic fungi, antifungal activity, antibacterial activity

## Abstract

Two endophytic fungi *Trichoderma afroharzianum* (HP-3) and *Alternaria alstroemeriae* (HP-7) were isolated and purified from the fresh root of *Dryopteris crassirhizoma*. Chemical investigation of the two fungi resulted in the isolation of two new phenols 2,4-dihydroxy-3-farnesyl-5-methoxy benzoic acid (**1**) and 2-hydroxyphenethyl 2-phenylacetate (**2**), together with 22 known compounds. Their structures were elucidated by NMR, UV, IR, HRESIMS, and comparison to the literature data. Compounds **15** and **16** showed significant antibacterial activity against *Micrococcus lysodeikticus* with MIC value of 6.25 μg/mL, while **8** and **14** displayed moderate inhibitory activities against several plant pathogenic fungi and clinically important bacterial strains. This is the first study to report the isolation, identification, and antimicrobial properties of metabolites from endophytic fungi of *D. crassirhizoma*. Our findings may provide lead compounds for the development of new antibacterial agents.

## 1. Introduction

Endophytes belong to the mitotic and meiotic ascomyces, which parasitize asymptomatically in healthy tissues below the epidermal cell layer of plants. During the long period of coexistence and evolution, mutualistic relationships have been established between endophytic fungi and host plants. The fungi promote the growth of host plants and enhance their resistance to biotic and abiotic stresses by accumulation of secondary metabolites such as terpenoids, alkaloids, phenols, lignans, and steroids, some of which can be used as drugs or lead compounds [1,2]. Podophyllotoxin, produced by endophytes from *Nothapotydes foetida*, *Podophyllum peltatum*, and *P. emodi*, is a valuable natural product as a precursor to three anticancer drugs, etoposide, teniposide, and etoposide phosphate [3]. In addition, compounds fusarubin, chetomin, and chaetocochin C isolated from plant endophytic fungi were reported to have significant in vitro antimycobacterial activity and could be developed as potential drugs against resistant mycobacterial infections [4].

The rhizome of *Dryopteris crassirhizoma* is a well-known Chinese herbal medicine used to treat parasitic infestation and viral diseases [5]. So far, there is no report on the chemical constituent or biological activity from endophytic fungus of *D. crassirhizoma*. Herein, our efforts with the chemical constituents of endophytic fungi *Trichoderma afroharzianum* and *Alternaria alstroemeriae* (HP-3 and HP-7) isolated from the rhizome of *D. crassirhizoma*, led to the isolation of two new (**1** and **2**) and 22 known (**3**~**24**) compounds. In this paper, we mainly describe their structural identification and antimicrobial activities in vitro.

## 2. Results and Discussion

### 2.1. Structure Elucidation of the Isolated Compounds

**Figure 1:**
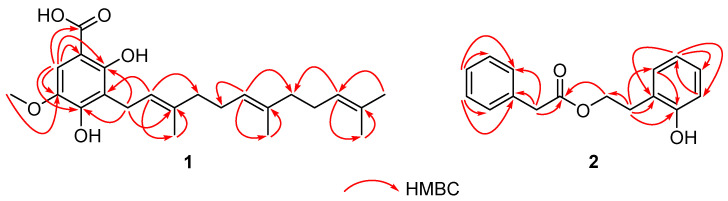
The key HMBC (red arrows) correlations of **1** and **2**.

**Figure 2:**
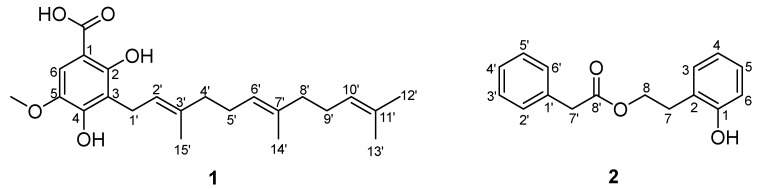
The structures of compounds **1** and **2**.

Compound **1** was obtained as a white powder and the molecular formula was determined as C_23_H_32_O_5_ based on the HRESIMS ion at *m*/*z* 411.2144 [M + Na]^+^ (calcd for 411.2142). The ^1^H and ^13^C NMR spectra (CDCl_3_) displayed the characteristic signals of three isopentenyl units at *δ*_H_ 3.41 (2H, d, 7.2)/*δ*_C_ 22.1 (C-1′); 5.26 (1H, t, 7.2)/121.3 (C-2′); 5.07 (2H, m)/124.2, 124.4 (C-6′, C-10′); 1.93, 1.99 (4H, m)/39.7, 39.8 (C-4′, C-8′); 2.00, 2.06 (4H, m)/26.7 (C-5′, C-9′); 1.66 (3H, s)/25.7 (C-12′), 1.58 (3H, s)/17.7 (C-3′); 1.56 (3H, s)/16.0 (C-14′); 1.78 (3H, s)/16.2 (C-15′); 135.9, 2×134.9 (C-3′, C-7′, C-11′) (Table 1). Additional signals observed in the 1D NMR data at *δ*_H_ 7.17 (1H, s)/*δ*_C_ 107.8 (C-6), *δ*_C_ 101.6 (C-1), 157.8 (C-2), 115.6 (C-3), 151.3 (C-4), and 139.8 (C-5) suggested the presence of pentasubstituted benzene. The NMR spectra of **1** and 2,4-dihydroxy-3-farnesyl-5-methoxy benzoic acid [6] were very similar, except for an additional hydroxyl group located at C-2 in **1**. This was confirmed by HMBC correlations from H_2_-1′ (*δ*_H_ 3.41) to C-2 (*δ*_C_ 157.8) and C-4 (*δ*_C_ 151.3), from OCH_3_ (*δ*_H_ 3.87) to C-5 (*δ*_C_ 139.8), and from H-6 (*δ*_H_ 7.17) to C-1 (*δ*_C_ 101.6), C-2 (*δ*_C_ 157.8), C-3 (*δ*_C_ 115.6), and C-4 (*δ*_C_ 151.3). (Figure 1) Thus, the structure of compound **1** was confirmed as 2,4-dihydroxy-3-farnesyl-5-methoxy benzoic acid. (Figure 2)

Compound **2**, a white powder, was found to possess the molecular C_16_H_16_O_3_ from the HRESIMS pseudo molecular ion [M + Na]^+^ at *m*/*z* 279.0990 (calcd. 279.0992), with 9 degrees of unsaturation. The ^1^H NMR and ^13^C NMR data (CDCl_3_) of **2** revealed the presence of a phenylacetyl unit [*δ*_H_ 7.25 (2H, m)/*δ*_C_ 129.3 (C-2′, C-6′), 7.31 (2H, m)/128.7 (C-3′, C-5′), 7.27 (1H, m)/127.2 (C-4′), 3.64 (2H, s)/41.4 (C-7′), and 172.0 (C-8′)] supported by HMBC correlations (H-7′/C-1′, C-6′, and C-8′), and a 2-hydroxyphenyl ester unit [*δ*_H_ 7.05 (1H, d, *J* = 7.6 Hz)/*δ*_C_ 130.9 (C-3), 6.84 (1H, t, *J* = 7.6 Hz)/*δ*_C_ 120.8 (C-4), 7.13 (1H, t, *J* = 7.6 Hz)/*δ*_C_ 128.2 (C-5), 6.80 (1H, d, *J* = 7.6 Hz)/*δ*_C_ 115.9 (C-6), 2.93 (2H, t, *J* = 6.7 Hz)/*δ*_C_ 29.9 (C-7), 4.29 (2H, t, *J* = 6.7 Hz)/*δ*_C_ 64.8 (C-8), 154.3 (C-1), and 123.5 (C-2)] proved by HMBC correlations (H-7/C-1, C-2, C-3, and C-8) (Figure 1). Combined with the molecular formula information, HMBC correlations of H-8/C-8′ verified the linkage of the two units via an ester bond, and the structure of **2** was defined as 2-hydroxyphenethyl 2-phenylacetate. (Figure 1 and Figure 2)

**Figure 3:**
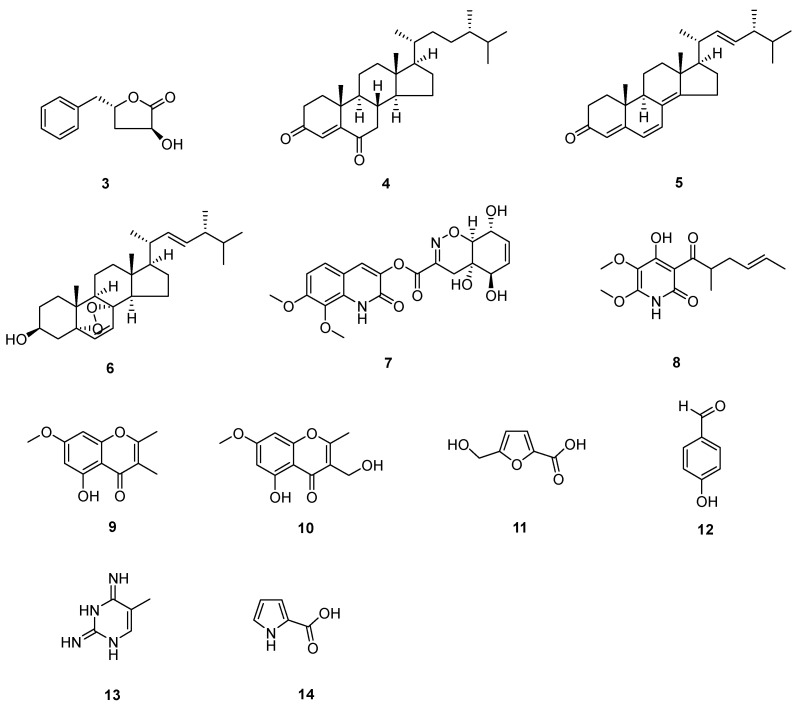
Compounds from *T. afoharzianum*.

**Figure 4:**
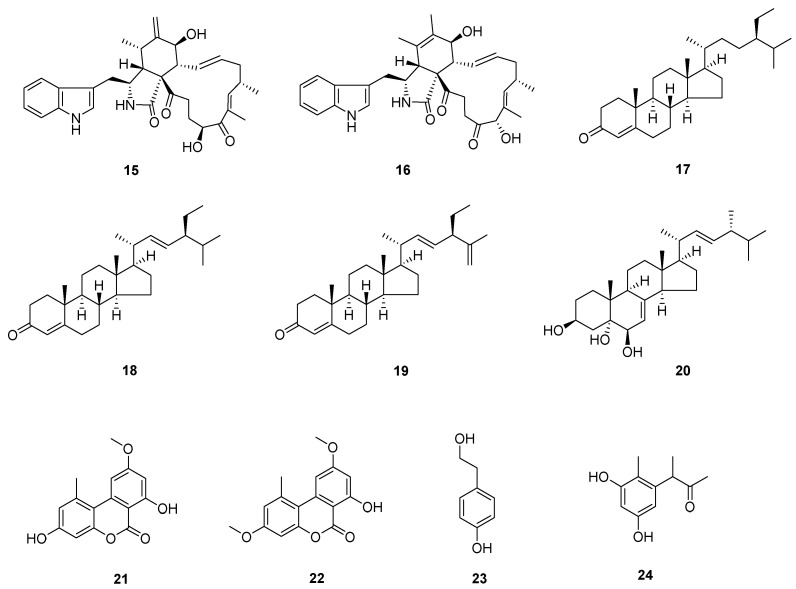
Compounds from *A*. *alstroemeriae*.

The known compounds (Figure 3 and Figure 4) *trans*-harzialactone A (**3**) [7], stigmast-4-ene-3,6-dione (**4**) [8], ergosta-4,6,8(14),22-tetraen-3-one (**5**) [9], (22*E*)-5*α*,8*α*-epidioxyergosta-6,22-dien-3*β*-ol (**6**) [10], penicillazine (**7**) [11], harzianopyridone (**8**) [12], 5-hydroxy-2,3-dimethyl-7-methoxychromone (**9**) [13], 5-hydroxy-3-hydroxymethyl-2-methyl-7-methoxychromone (**10**) [13], 5-hydroxymethyl-2-furancarboxylic acid (**11**) [14], 4-hydroxybenzaldehyde (**12**) [15], 5-methylpyrimidine-2,4-diamin (**13**) [16], pyrrole-2-carboxylic acid (**14**) [16], chaetoglobosin F (**15**) [17], chaetoglobosin B (**16**) [18], *β*-sitostenone (**17**) [19], (22*E*, 24*S*)-stigmasta-4,22-dien-3-one (**18**) [20], (22*E*, 24*S*)-stigmasta-4,22,25-trien-3-one (**19**) [21], 5*β*-cholestane-3*β*,5,6*β*-triol (**20**) [22], alternariol 9-methyl ether (**21**) [23], alternariol 4,10-*O*-dimethyl ether (**22**) [24], 4-(2-hydroxyethyl) phenol (**23**) [25], and 3,3′,5′-dihydroxy-2′-methylphenyl-2-butanone (**24**) [26] were identified by comparison of their spectral data with the literature values.

### 2.2. *Antifungal* Activity of the Isolated Compounds

Antifungal activity of compounds **1**~**24** was evaluated using four plant pathogenic fungi, including *Verticillium dihliae* Kleb, *Rhizoctonia solani*, *Sclerotinia sclexotiorum*, and *Physalospora pixicolg* Nose, following the procedures described in the literature [27,28]. Ketoconazole was employed as the reference standard in the experiment. The results (Table S1 in Supplementary Materials) revealed that compound **8** displayed moderate inhibitory activity against three plant pathogenic fungi (*V. dahliae* Kleb, *S. sclexotiorum*, and *P. pixicolg* Nose) with MIC values of 50, 50, and 12.5 μg/mL, respectively.

### 2.3. Antibacterial Activity of the Isolated Compounds

Compounds **1**~**24** were tested for their antibacterial activity against clinically important bacterial strains including *M. lysodeticus*, *Bacillus subtilis*, *M*. *luteus*, *Salmonella typhi*, *Alternaria longipes*, and *Staphylococcus aureus*. The assay was performed using a previously described method, with ciprofloxacin utilized as the reference standard [27,28]. Compounds **15** and **16** exhibited significant inhibitory effects (Table S2 in Supplementary Materials) against *M*. *lysodeikticus* with MIC value of 6.25 μg/mL. And **14** showed moderate inhibitory activities against *M. lysodeikticus* and *M*. *luteus* with MIC values of 25 and 50 μg/mL, respectively.

## 3. Materials and Methods

### 3.1. Fermentation, Extraction, and Isolation

**Figure 5:**
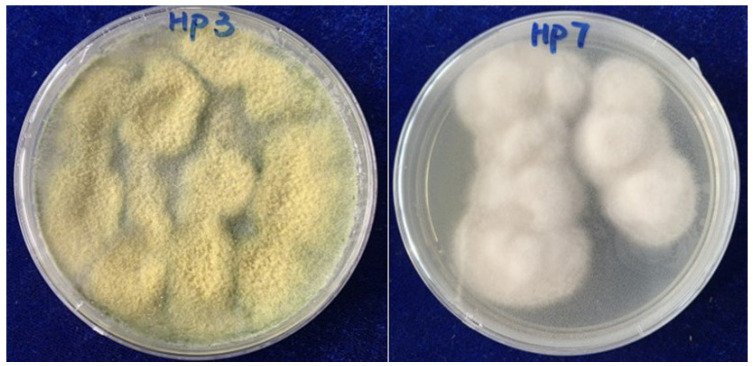
The plates of *T. afroharzianum* strain HP-3 and *A. alstroemeriae* strain HP-7.

The study material *T*. *afroharzianum* strain HP-3 and *A*. *alstroemeriae* strain HP-7 (Figure 5) were isolated from the root of *D. crassirhizoma*, collected from Fushun, Liaoning province. The endophytic fungi (HP-3 and HP-7) were identified by internal transcribed spacer (ITS) sequencing by Chongqing Biomedicine Biotechnology Co., Ltd, Chongqing, China. The fungi were grown on PDA for 5 days, and DNA templates were prepared by the SDS extraction method [29]. Briefly, mycelium were scraped off from culture plates and transferred into a centrifuge tube. Mycelium were mixed with 500 μL of buffer [50 mM of EDTA (PH 8.0), 100 mM of Tris-HCl (PH 8.0), 500 mM of NaCl, and SDS (20%)] and incubated at 65 °C for 35 min. Then 100 μL of chloroform was mixed into the tube and allowed to stand for 5 min, then centrifuged at 12,000 rpm for 15 min. The aqueous extraction layer (200~400 μL) was transferred into a new tube, followed by the addition of an equal volume of isopropyl alcohol. The tube was gently inverted and left to stand for 10 minutes, then centrifuged at 12,000 rpm for 10 min at RT to precipitate DNA. The DNA pellet was washed with 75% ethanol twice and dried, and their concentration and quality were measured by using UV spectrophotometer at 230, 260, and 280 nm. The ITS regions of the fungus were amplified with the ITS primers ITS-F (TCCGTAGGTGAACCTGCGG) and ITS-R (TCCTCCGCTTATTGATATGC) using the polymerase chain reaction (PCR). The PCR conditions used were as follows: initial denaturation at 95 °C for 3 min, followed by 35 cycles of 95 °C for 10 s, 55 °C for 30 s, 72 °C for 10 s, and a final extension at 72 °C for 5 min. The reaction mixture contained 22 μL of GoldenStar T6 Super PCR Mix, 0.5 μL of each primer (10 μΜ), 2 μL of template DNA, and 25 μL of ddH_2_O. After electrophoresis at 120 V for 20 min, the amplified products were visualized on 1% (*w*/*v*) agarose gel to confirm the presence of a single amplified band (Figure S34 in Supplementary Materials). Then the band due to the PCR products was isolated from the gel slice using the DNA gel purification kit (Tsingke, Beijing, China) according to the manufacturer’s protocol. PCR products were sequenced using electrophoretic sequencing on an ABI 3730XL DNA analyzer (Applied Biosystems, Foster City, CA, USA). The sequences were matched against the nucleotide nucleotide database (BLASTn) of the National Center for Biological Information (NCBI) for final identification of the endophytic isolate. The ITS sequence of strain HP-3 showed high homologies of 99% (in 564 bp) to *Trichoderma afroharzianum* (CBS 124620), while the sequence of HP-7 showed high homologies of 99% (in 518 bp) to *Alternaria alstroemeriae* (CBS 118809) (Figures S35 and S36 in Supplementary Materials). The endophytic fungi were stored at the Laboratory of the Department of Materials and Chemistry, Yibin University. The HP-3 and HP-7 strains were mass cultivated in 500 mL sterilized Erlenmeyer flasks, each containing rice (100 g), potato extract broth medium (100 mL), and the seed culture (20.0 mL) at room temperature for 60 days. The two solid fermented materials (200 flasks each strain) were extracted three times with equivoluminal EtOAc to obtain extracts HP-3 (51 g) and HP-7 (113 g), respectively. The HP-3 extracts were then subjected to silica gel CC (200~300 mesh) with petroleum ether PE/EtOAc (from 100:0 to 0:100) to obtain 13 fractions (A~M). Fraction B was rechromatographed on silica gel CC eluted with PE/EtOAc (from 15:0 to 1:1) to yield subfractions (Fr.B1~Fr.B6).

Compound **9** (145 mg) was precipitated from subfraction B2. Fr. D (0.50 g) was fractioned over Sephadex LH-20 column (CH_2_Cl_2_/MeOH = 1:1) and semi-preparative HPLC (MeOH/H_2_O = 95:5), to obtain compounds **4** (1.6 mg) and **5** (20 mg). Fr. F (9.0 g) was separated on silica gel column eluted with CH_2_Cl_2_/MeOH (20:1) to provide subfractions (F1~F4). Fr. F3 (0.66 g) was fractioned over Sephadex LH-20 column (CH_2_Cl_2_/MeOH = 1:1) and semi-preparative HPLC (MeOH/H_2_O = 62:38), to obtain compounds **2** (1.1 mg) and **8** (13mg, 0.00025%). Fr. G (2.5 g) was separated on silica gel column eluted with CH_2_Cl_2_/MeOH = 20:1 to provide subfractions (G1~G6). Compound **10** (20.2 mg) was precipitated from Fr. G2. The mother liquor of Fr. G2 was fractioned over Sephadex LH-20 column (CH_2_Cl_2_/MeOH = 1:1) and semi-preparative HPLC (MeOH/H_2_O = 65:35) to obtain compounds **3** (15 mg), **6** (10 mg), **12** (4 mg), and **14** (2.7 mg). Fr. L was submitted to silica gel column (CH_2_Cl_2_/EtOAc = 1:1), Sephadex LH-20 column (CH_2_Cl_2_/MeOH = 1:1), and semi-preparative HPLC (MeOH/H_2_O = 70:30) to afford **7** (23.7 mg), **11** (17.0 mg), and **13** (16.5 mg).

The HP-7 extracts were applied to CC (200~300 mesh), eluting with PE/EtOAc (1:0→0:1 gradient), to yield 11 fractions (A~K). Compound **21** (65 mg) was precipitated from subfractions Fr. B. Fraction D (9.0 g) was subjected to silica gel CC, eluting with PE/EtOAc (20:1) to yield five fractions (D1~D5). Fr. D2 (0.43 g) was fractioned over Sephadex LH-20 column (CH_2_Cl_2_/MeOH = 1:1) and semi-preparative HPLC (MeOH/H_2_O = 88:12), to obtain compound **17** (11 mg). Fraction D3 (2.0 g) was separated on Sephadex LH-20 column (CH_2_Cl_2_/MeOH = 1:1) and semi-preparative HPLC with 95% MeOH, to yield **22** (3.1 mg), **18** (4.4 mg), and **19** (3.0 mg). Compound **24** (4.6 mg) was obtained from Fr. G by repeated Sephadex LH-20 column (CH_2_Cl_2_/MeOH = 1:1) and semi-preparative HPLC (MeOH/H_2_O = 55:45). Fr. I (3.0 g) was fractioned over Sephadex LH-20 column (CH_2_Cl_2_/MeOH = 1:1) and semi-preparative HPLC (85% MeOH) to obtain compound **1** (6.3 mg). After separation by Sephadex LH-20 column (CH_2_Cl_2_/MeOH = 1:1) and then preparative HPLC (45% MeOH), **23** (6.6 mg) was isolated from Fr. J. Fr. K (4.1 g) was separated on MCI gel column (30% to 100% MeOH) and semi-preparative HPLC with 35% MeOH, to yield compounds **15** (20 mg, 0.00018%), **16** (2.0 mg, 0.000018%), and **20** (45.0 mg). 

2,4-Dihydroxy-3-farnesyl-5-methoxy benzoic acid (**1**): white powder; UV (MeOH) *λ*_max_ (logε): 196 (4.7), 253 (3.9), 310 (3.8) nm; IR (KBr) *ν*_max_: 3492, 2925, 1617, 1457, 1236 cm^–1^; ^1^H and ^13^C NMR data see Table 1.

2-Hydroxyphenethyl 2-phenylacetate (**2**): white powder; UV (MeOH) *λ*_max_ (log*ε*): 195 (4.7), 274 (3.9) nm; IR (KBr) *ν*_max_: 3434, 2922, 2851, 1719, 1456 cm^–1^; ^1^H and ^13^C NMR data see Table 1.

### 3.2. Assay of Anti-Plant Pathogenic Fungi Activity

Using the microbroth dilution method [27,28], four plant pathogenic fungi (*V. dahliae Kleb, R. solani, S. sclexotiorum,* and *P. pixicolg* Nose) were used to evaluate the antifungal activity of all isolated compounds (**1**~**24**). These fungi were cultured in potato dextrose agar (PDA) at 28 °C for 72 h to prepare a suspension solution of fungi (104 mycelia fragments/mL). All test compounds and ketoconazole (positive control) were prepared in DMSO at a concentration of 1 mg/mL. The 180 μL of fungi suspension and 20 μL of the above-prepared solutions were added into 96-well flat plates in triplicate. Test compounds and ketoconazole were diluted using 2-fold serial dilution method, resulting in concentrations ranging from 100 to 0.78 μg/mL. Additionally, the medium containing 1% DMSO was used as blank control. The MIC was determined by incubating the plate. The MIC values were defined as the lowest test concentration with no obvious growth after the incubation (28 °C, 72 h).

### 3.3. Assay of Antibacterial Activity In Vitro

All isolated compounds (**1**~**24**) were evaluated for their antibacterial activity against *M. lysodeikticus*, *B. cereus*, *M. luteus*, *Salmonella typhimurium*, *P. aeruginosa*, and *S. aureus* bacteria. Targeted microbes were cultivated in Luria Bertani (LB) medium at 37 °C for 24 h to prepare the suspension solution (1 × 10^6^ CFU/mL) for tests. Compounds and ciprofloxacin (positive control) were prepared in DMSO (1 mg/mL) as stock solution. Bacterial suspension (180 μL) and stock solutions (20 μL) were added to 96-well flat plates in triplicate. The final concentrations of test compounds and ciprofloxacin were 100, 50, 25, 12.5, 6.25, 3.12, 1.56, 0.78 μg/mL in medium. After incubation at 37 °C for 24 h, the MICs were defined as the lowest test concentration that showed no obvious growth.

## 4. Conclusions

This is the first report about the chemical constituents of endophytic fungi isolated from *D. crassirhizoma*. A total of 24 compounds including two new ones were isolated from the solid fermentation of the fungi *T. afroharzianum* and *A. alstroemeriae*. In the in vitro bioassays, compounds **15** and **16** significantly inhibited *M*. *lysodeikticus* with MIC value of 6.25 μg/mL. Compound **8** displayed inhibitory activity against three plant pathogenic fungi (*V. dahliae* Kleb, *S. sclexotiorum*, and *P. pixicolg* Nose) with MIC values of 12.5~50 μg/mL. In addition, **14** showed moderate inhibitory activities against *M. lysodeikticus* and *M*. *luteus* with MIC values of 25 and 50 μg/mL, respectively. The cytochalasin-type alkaloids Chaetoglobosins F (**15**) and B (**16**), previously isolated from the genus *Chaetomium*, *Alternaria*, *Penicillium*, etc, were found to have a wide range of biological activities, such as cytotoxic, immunomodulatory, antimicrobial, antioxidative, and neuroprotective properties [30,31,32,33,34,35]. Notably, Andrew and co-workers found that chaetoglobosin B (**16**) possessed antibacterial activity towards methicillin-resistant *Staphylococcus aureus* and *Mycobacterium tuberculosis* H37Ra (IC_50_: 85 and 22 µM) [36]. In our paper, is the first report that chaetoglobosins F and B (**15** and **16**) both possessed strong inhibitory effects against *M*. *lysodeikticus*. This indicated the possible application of cytochalasan alkaloids in developing new antibacterial agents.

## Figures and Tables

**Table 1:** ^1^H NMR (600 MHz) and ^13^C NMR (150 MHz) data for compounds **1** and **2** ^a^.

1			2		
No.	*δ*_H_ (mult., *J* in Hz)	*δ*_C_ (mult.)	No.	*δ*_H_ (mult., *J* in Hz)	*δ*_C_ (mult.)
1		101.6, C	1		154.3, C
2		157.8, C	2		123.5, C
3		115.6, C	3	7.05, d, 7.6	130.9, CH
4		151.3, C	4	6.84, t, 7.6	120.8, CH
5		139.8, C	5	7.13, t, 7.6	128.2, CH
6	7.17, s	107.8, CH	6	6.80, d, 7.6	115.9, CH
1′	3.41, d, 7.2	22.1, CH_2_	7	2.93, t, 6.7	29.9, CH_2_
2′	5.26, t, 7.2	121.3, CH	8	4.29, t, 6.7	64.8, CH_2_
3′		135.9, C	1′		133.6, C
4′	1.93, 1.99, m	39.8, CH_2_	2′	7.25, m	129.3, CH
5′	2.00, 2.06, m	26.7, CH_2_	3′	7.31, m	128.7, CH
6′	5.07, overlapped	124.2, CH	4′	7.27, m	127.2, CH
7′		134.9, C	5′	7.31, m	128.7, CH
8′	1.93, 1.99, m	39.7, CH_2_	6′	7.25, m	129.3, CH
9′	2.00, 2.06, m	26.7, CH_2_	7′	3.64, s	41.4, CH_2_
10′	5.07, overlapped	124.4, CH	8′		172.0, C
11′		134.9, C			
12′	1.66, s	25.7, CH_3_			
13′	1.58, s	17.7, CH_3_			
14′	1.56, s	16.0, CH_3_			
15′	1.78, s	16.2, CH_3_			
-OCH_3_	3.87, s	56.3, CH_3_			

^a^ Recorded in CDCl_3_.

## Data Availability

Data is contained within the article or Supplementary Materials.

## Supplementary Materials

The following supporting information can be downloaded at: https://www.mdpi.com/article/10.3390/molecules28248043/s1, general experimental procedures; Table S1: antifungal activities; Table S2: antibacterial activities; Figures S1~S33: HRESIMS, and 1D, 2D NMR spectra of compounds **1**~**24**; Figure S34: gel electrophoresis of 16S rRNA gene amplicons; Figures S35 and S36: sequence data analysis of HP-3 and HP-7.
