# Supplementary material for: Two New Compounds from the Endophytic Fungi of Dryopteris crassirhizoma and Their Antimicrobial Activities
Source: Molecules. 2023 Dec 12;28(24):8043. doi: 10.3390/molecules28248043 (PMC10745856; doi:10.3390/molecules28248043)
Supplement: Supplementary file 1 [file molecules-28-08043-s001.zip › molecules-2673697-supplementary.pdf]

Two New Compounds from the Endophytic Fungi of *Dryopteris crassirhizoma* and Their Antimicrobial Activities

Ping Hai[1,2], Yuan Gao[2], Lian Yang[1], Nie Chen[2], Haiyan Jia[2], MengdieWang[2], Huan Li[2], Wenli Jiang[2], Jian Yang[3,*], Rongtao Li[1,*]

[1] *Faculty of Life Science and Technology, Kunming University of Science and Technology, Kunming China*

[2] *Faculty of Materials and Chemical Engineering, Yibin University, Yibin China*

[3] *State Key Laboratory Breeding Base of Dao-di Herbs, National Resource Center for Chinese Materia Medical, China Academy of Chinese Medical Sciences, Beijing China*

*Corresponding author

E-mail addresses: lirt@kust.edu.cn (Rongtao Li); yangchem@nrc.ac.cn (Jian Yang).

Contents

1. General experimental procedures.. 3

2. Table S1 Antifungal activity of compounds 1~24 (MIC, μg/mL)................... 4

3. Table S2 Antibacterial activity of compounds 1~24 (MIC, μg/mL)............... 5

4. NMR and MS spectra of compound 1.. 6

5. NMR and MS spectra of compound 2..11

6. NMR spectrum of compounds 3~24. 14

Figure S34 The gel electrophoresis of 16S rRNA gene amplicons...................25

Figure S35 The sequence data analysis of HP-3..26

Figure S36 The sequence data analysis of HP-7..27

1. General experimental procedures

Optical rotations were measured with a Rudolph AUTOPOL VI polarimeter. UV spectra were obtained using an Agilent Cary60 spectrophotometer. IR spectra were obtained on a Bruker Tensor 27 FT-IR spectrometer with KBr pellets. NMR spectra were acquired with a Bruker Avance III 500 or 600 instrument at room temperature. ESI-MS and HR-ESI-MS were performed on an Agilent G6230 time-of-flight mass spectrometer. Silica gel (200–300 mesh) and Sephadex LH-20 (Amersham Biosciences, Sweden) were used for column chromatography. Semi-prep HPLC was performed on an AS20005 series (Hanbon, China) using a 5C18-AR-II column (5 μm, 10 × 250 mm, 3.0 mL/min, Nacalai Tesque, Japan), or a P3500 series (Dalian Elite, China) using a Sinochrom ODS-BP column (10 μm, 30 × 250 mm, 15 mL/min, Dalian Elite, China).

2. Table S1 Antifungal activity of compounds 1~24 (MIC, μg/mL)

Compound	Fungal pathogen			
	Verticillium dahliae Kleb	*Rhizoctonia solani*	*Sclerotinia sclexotiorum*	*Phsalospora pixicolg Nose*
**1**	>100	>100	>100	>100
**2**	>100	>100	>100	>100
**3**	>100	>100	>100	>100
**4**	>100	>100	>100	>100
**5**	>100	>100	>100	>100
**6**	>100	>100	>100	>100
**7**	>100	>100	>100	>100
**8**	50	>100	50	12.5
**9**	>100	>100	>100	>100
**10**	>100	>100	>100	>100
**11**	>100	>100	>100	>100
**12**	>100	>100	>100	>100
**13**	>100	>100	>100	>100
**14**	>100	>100	>100	>100
**15**	>100	>100	>100	>100
**16**	>100	>100	>100	>100
**17**	>100	>100	>100	>100
**18**	>100	>100	>100	>100
**19**	>100	>100	>100	>100
**20**	>100	>100	>100	>100
**21**	>100	>100	>100	>100
**22**	>100	>100	>100	>100
**23**	>100	>100	>100	>100
**24**	>100	>100	>100	>100
Ketoconazole	0.78	6.25	1.56	1.56

3. Table S2 Antibacterial activity of compounds 1~24 (MIC, μg/mL)

Compound	Bacterial pathogen					
	Micrococcus lysodeikticus	*Bacillus subtilis*	*Micrococcus luteus*	*Salmonella typhi*	*Alternaria longipes*	*Staphylococcus aureus*
**1**	>100	>100	>100	>100	>100	>100
**2**	>100	>100	>100	>100	>100	>100
**3**	>100	>100	>100	>100	>100	>100
**4**	>100	>100	>100	>100	>100	>100
**5**	>100	>100	>100	>100	>100	>100
**6**	>100	>100	>100	>100	>100	>100
**7**	>100	>100	>100	>100	>100	>100
**8**	>100	>100	>100	>100	>100	>100
**9**	>100	>100	>100	>100	>100	>100
**10**	>100	>100	>100	>100	>100	>100
**11**	>100	>100	>100	>100	>100	>100
**12**	>100	>100	>100	>100	>100	>100
**13**	>100	>100	>100	>100	>100	>100
**14**	25	>100	50	>100	>100	>100
**15**	6.25	>100	>100	>100	>100	>100
**16**	6.25	>100	>100	>100	>100	>100
**17**	>100	>100	>100	>100	>100	>100
**18**	>100	>100	>100	>100	>100	>100
**19**	>100	>100	>100	>100	>100	>100
**20**	>100	>100	>100	>100	>100	>100
**21**	>100	>100	>100	>100	>100	>100
**22**	>100	>100	>100	>100	>100	>100
**23**	>100	>100	>100	>100	>100	>100
**24**	>100	>100	>100	>100	>100	>100
Ciprofloxacin	0.78	0.78	0.78	0.78	0.78	0.78

4. NMR and MS spectra of compound 1

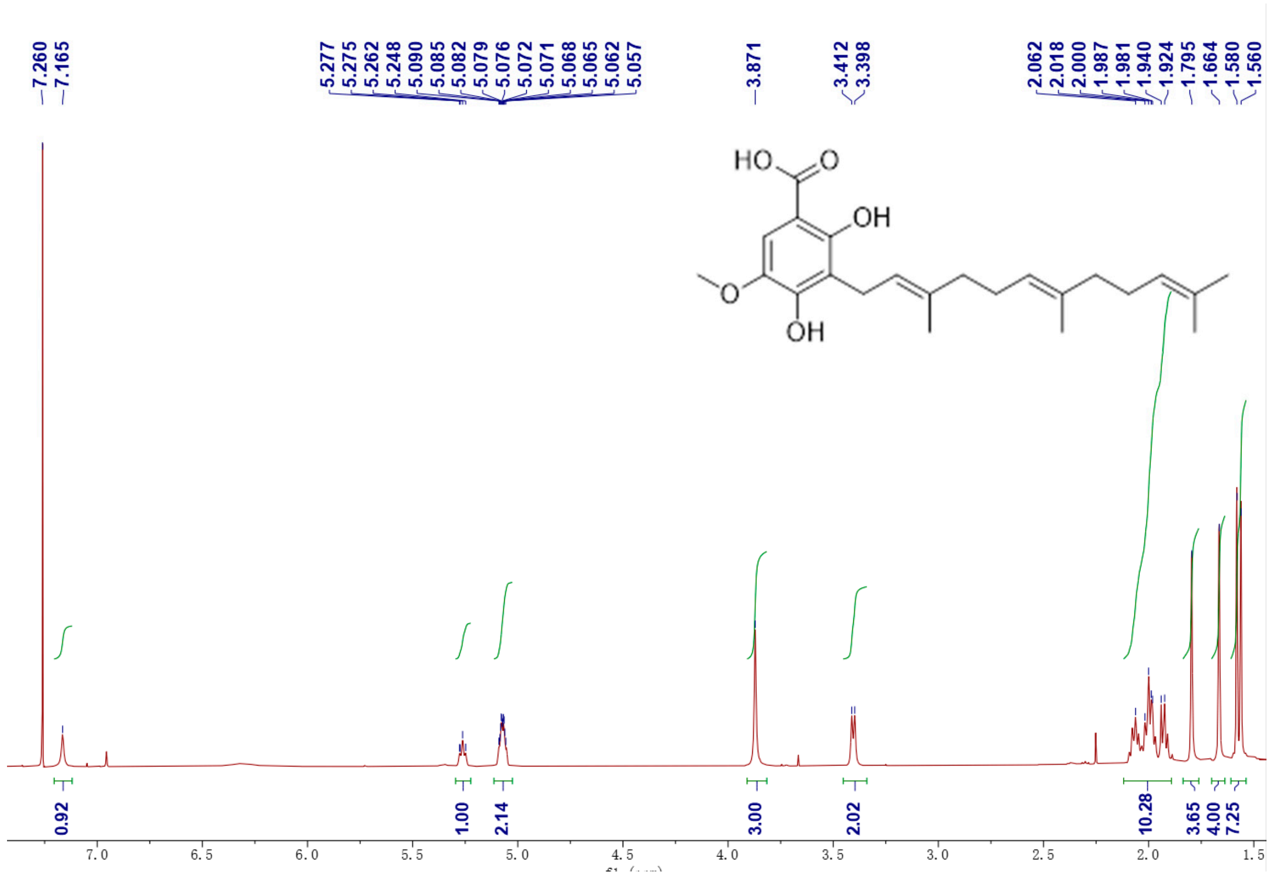

Figure S1 ^{1}H-NMR spectrum (500 MHz) of 1 in $CDCl_3$.

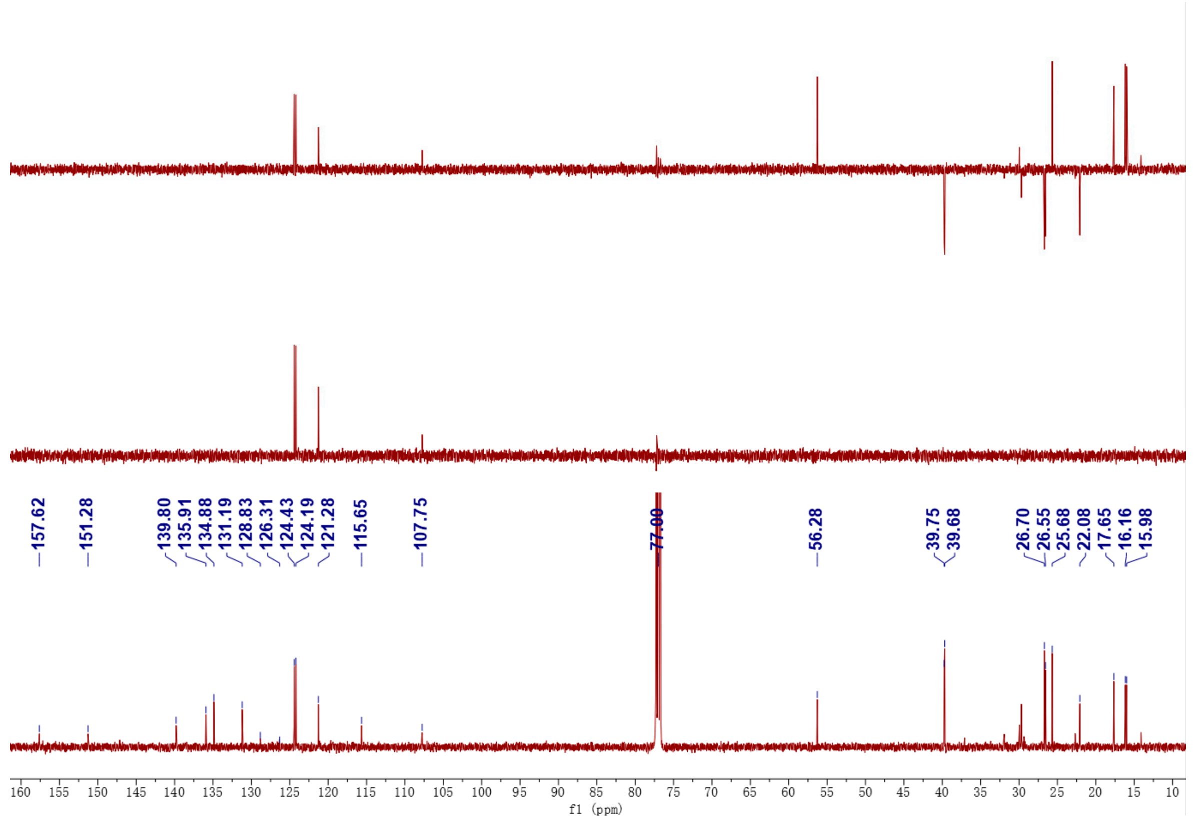

Figure S2 ^{13}C-NMR spectrum (125 MHz) of 1 in $CDCl_3$.

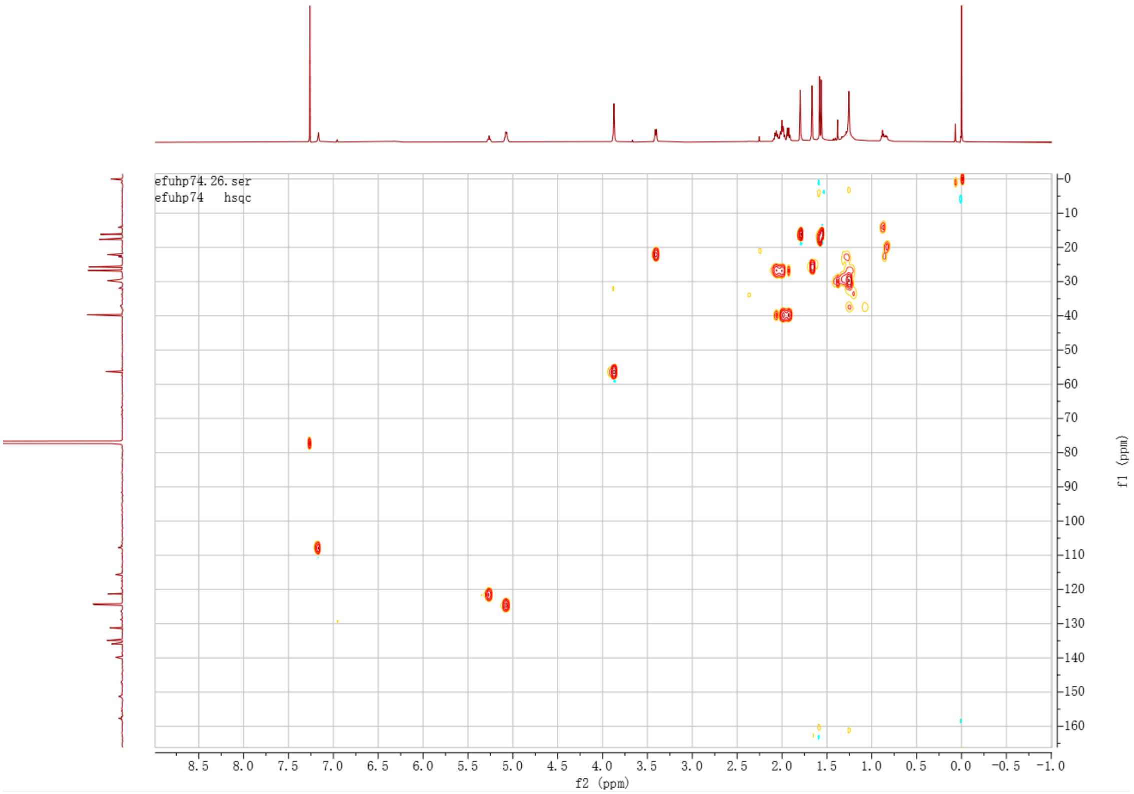

Figure S3 HSQC spectrum (500 MHz) of 1 in $CDCl_3$.

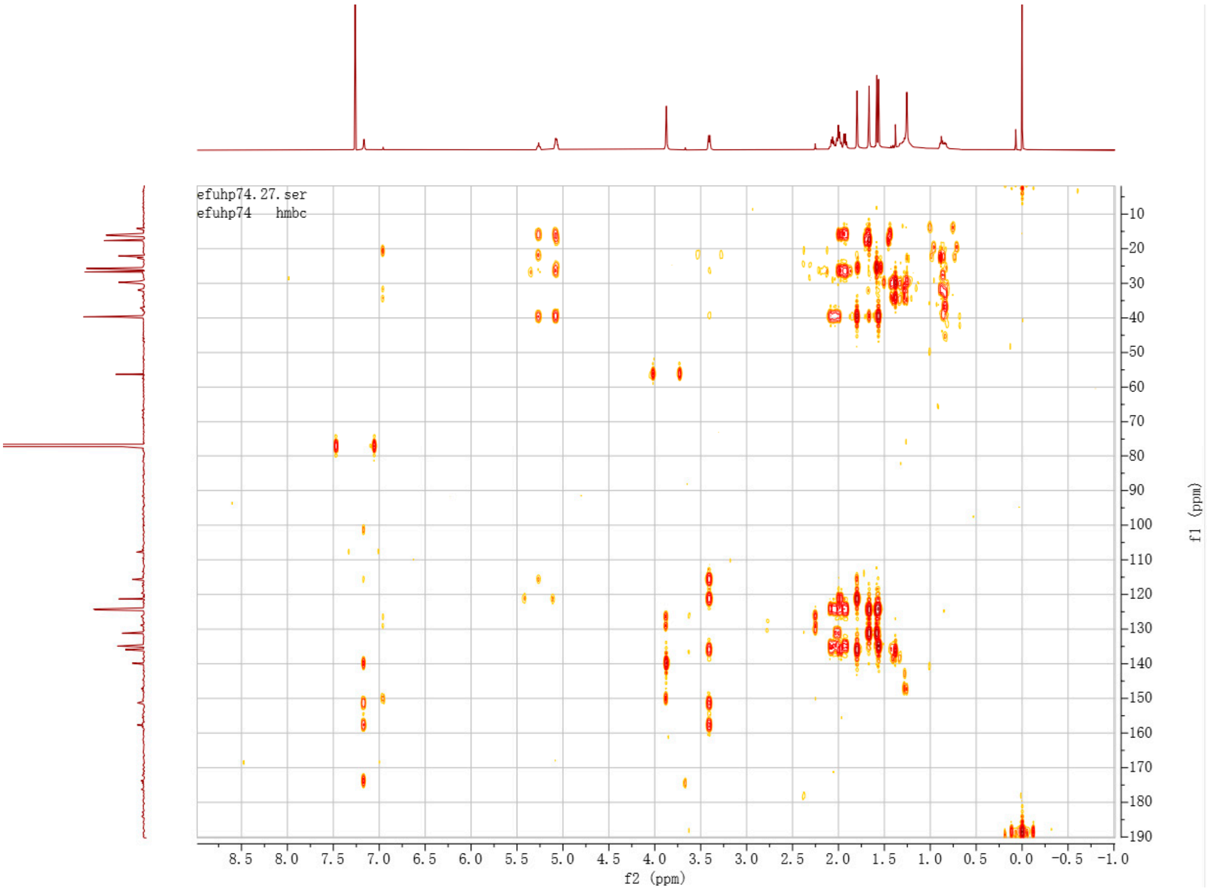

Figure S4 HMBC spectrum (500 MHz) of 1 in $CDCl_3$.

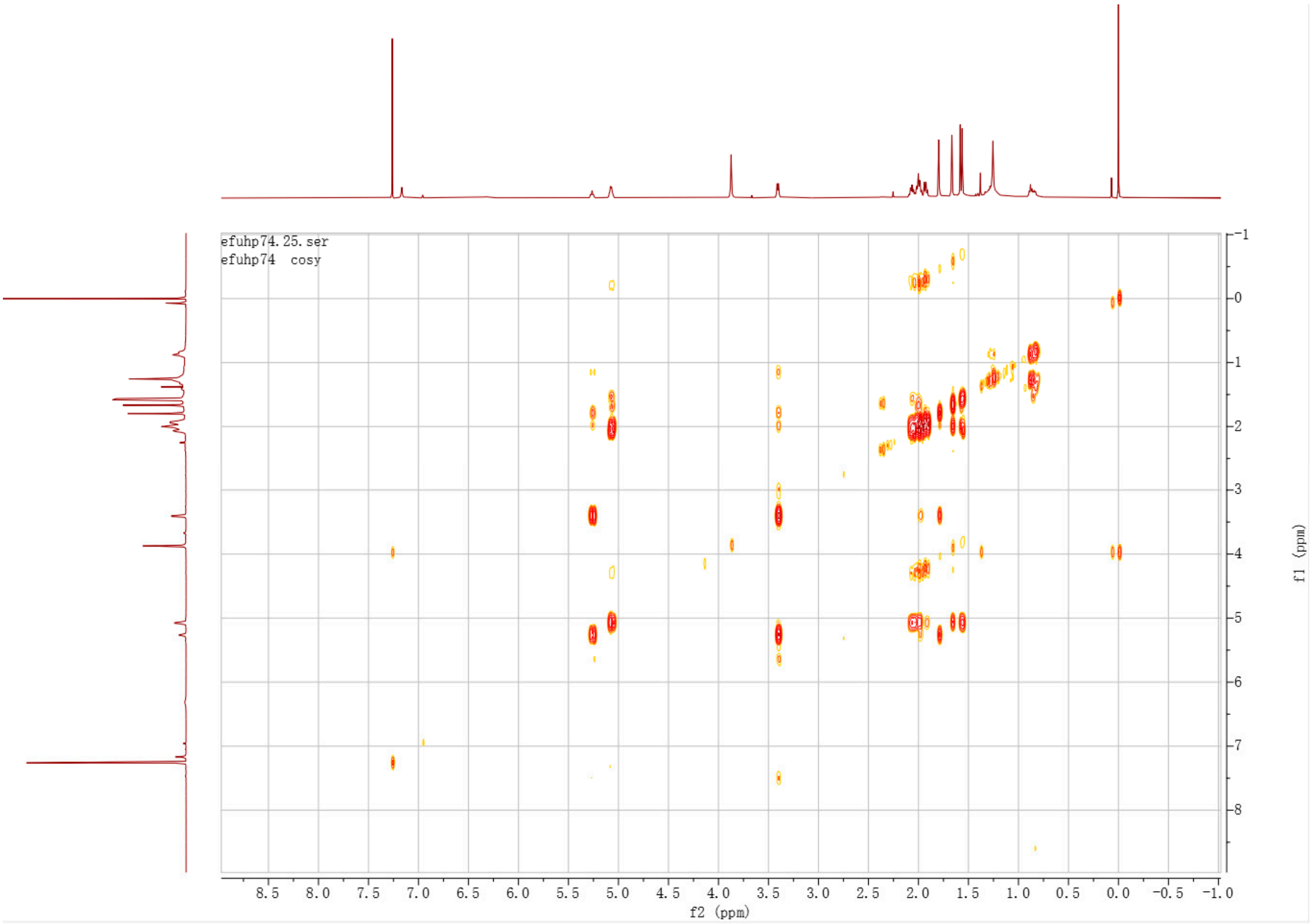

Figure S5 COSY spectrum (500 MHz) of 1 in $CDCl_3$.

Formula Predictor Report - Efuhp74.lcd Page 1 of 1

Data File: E:\DATA\2021\1101\Efuhp74.lcd

Elmt	Val.	Min	Max	Elmt	Val.	Min	Max	Elmt	Val.	Min	Max	Elmt	Val.	Min	Max	Use Adduct
H	1	5	100	F	1	0	0	Cl	1	0	0	Ag	1	0	0	Na
2H	1	0	0	Na	1	0	0	Co	2	0	0	I	3	0	0	
B	3	0	0	Mg	2	0	0	Cu	2	0	0	Ir	3	0	0	
C	4	5	50	Si	4	0	0	Se	2	0	0					
N	3	0	10	P	3	0	0	Br	1	0	0					
O	2	0	30	S	2	0	0	Pd	2	0	0					

Error Margin (ppm): 5
HC Ratio: unlimited
Max Isotopes: all
MSn Iso RI (%): 75.00

DBE Range: not fixed
Apply N Rule: yes
Isotope RI (%): 1.00
MSn Logic Mode: OR

Electron Ions: both
Use MSn Info: yes
Isotope Res: 10000
Max Results: 20

Event#: 1 MS(E+) Ret. Time : 0.467 -> 0.547 Scan# : 71 -> 83

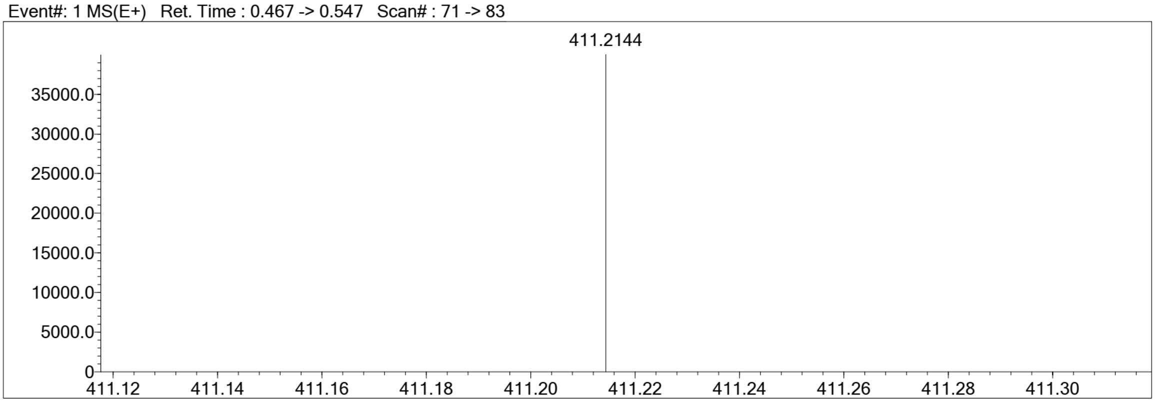

Measured region for 411.2144 m/z

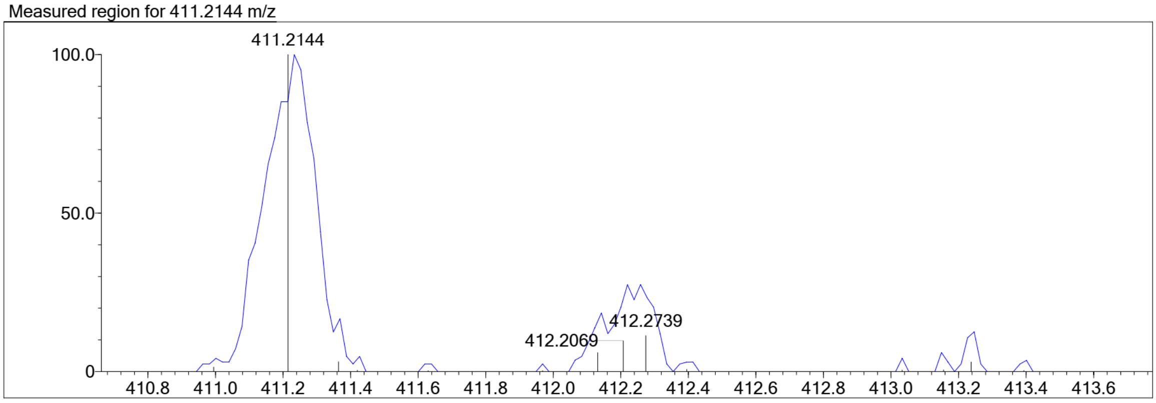

C23 H32 O5 [M+Na]+ : Predicted region for 411.2142 m/z

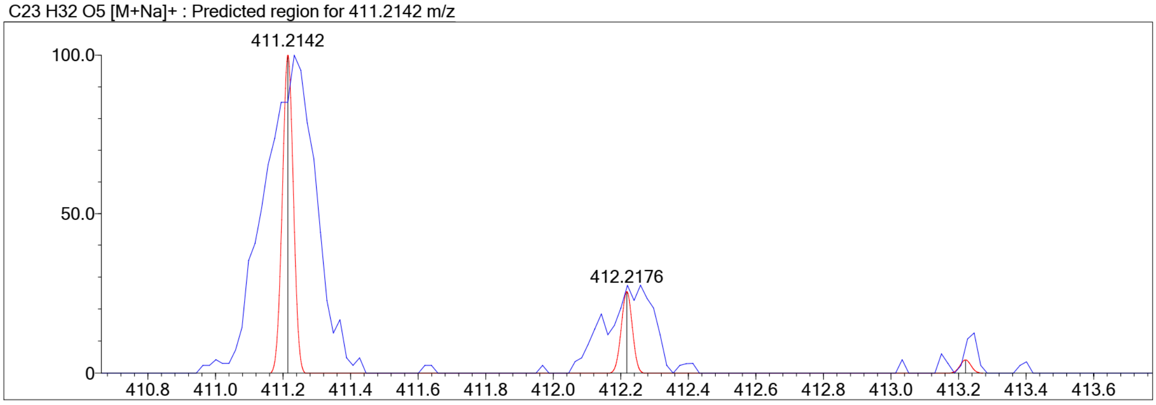

Formula (M)	Ion	Meas. m/z	Pred. m/z	Df. (mDa)	Df. (ppm)	DBE
C23 H32 O5	[M+Na]+	411.2144	411.2142	0.2	0.49	8.0

Figure S6 HRESIMS spectrum of 1.

5. NMR and MS spectra of compound 2

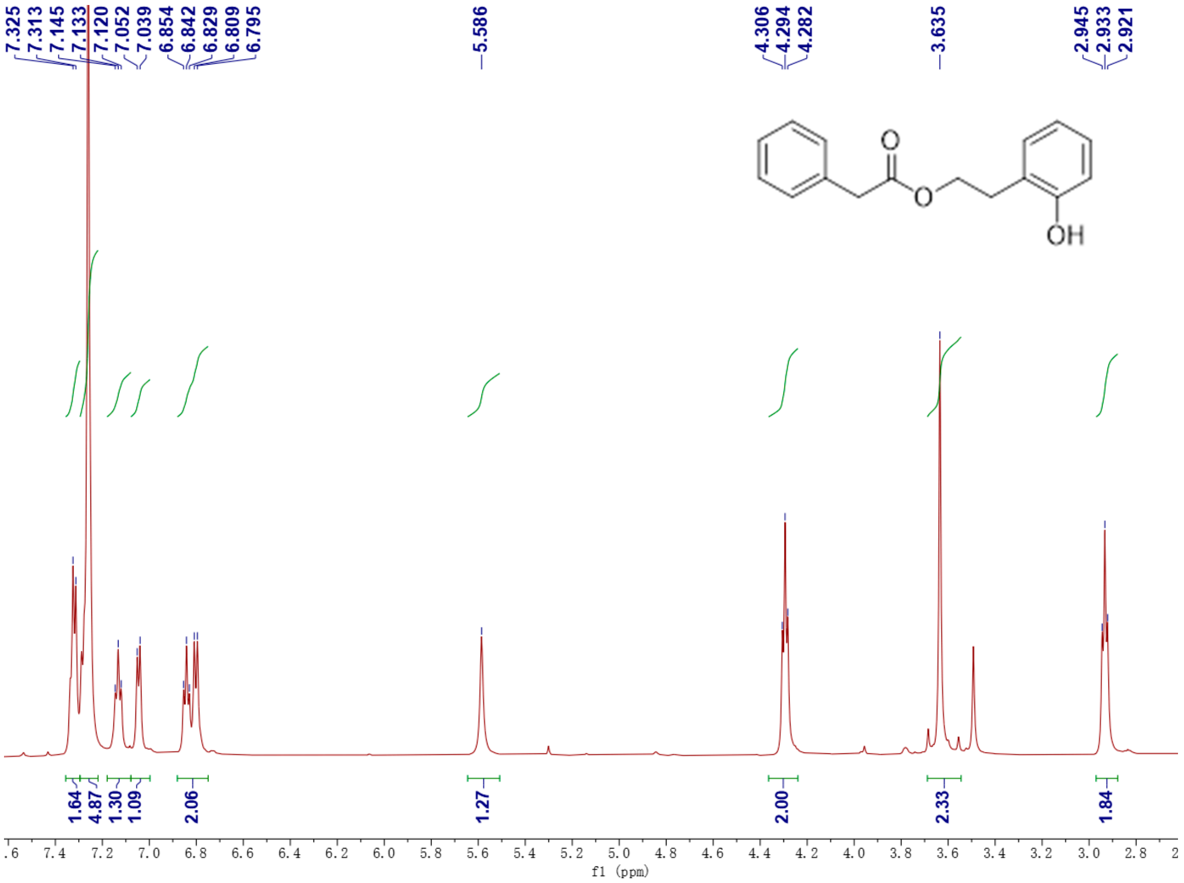

Figure S7 ^{1}H-NMR spectrum (600 MHz) of 2 in $CDCl_3$.

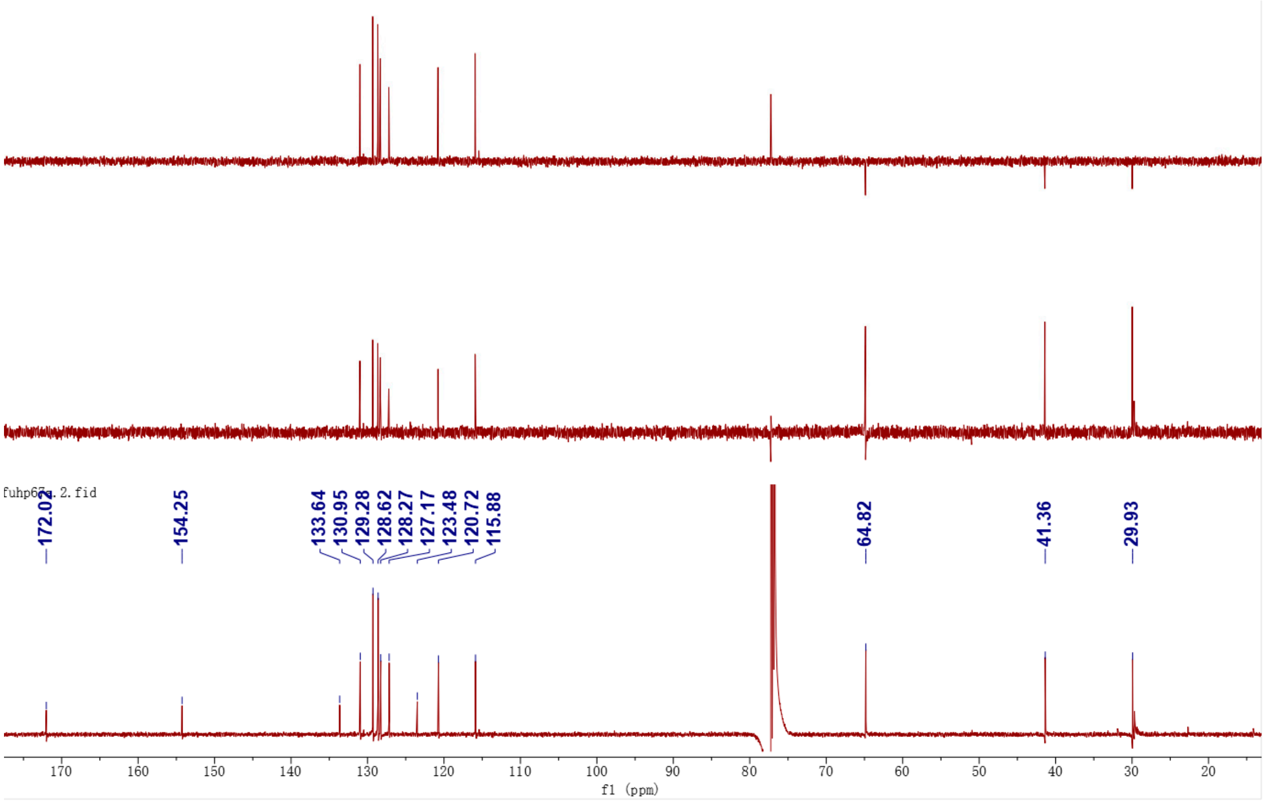

Figure S8 ^{13}C-NMR spectrum (150 MHz) of 2 in $CDCl_3$.

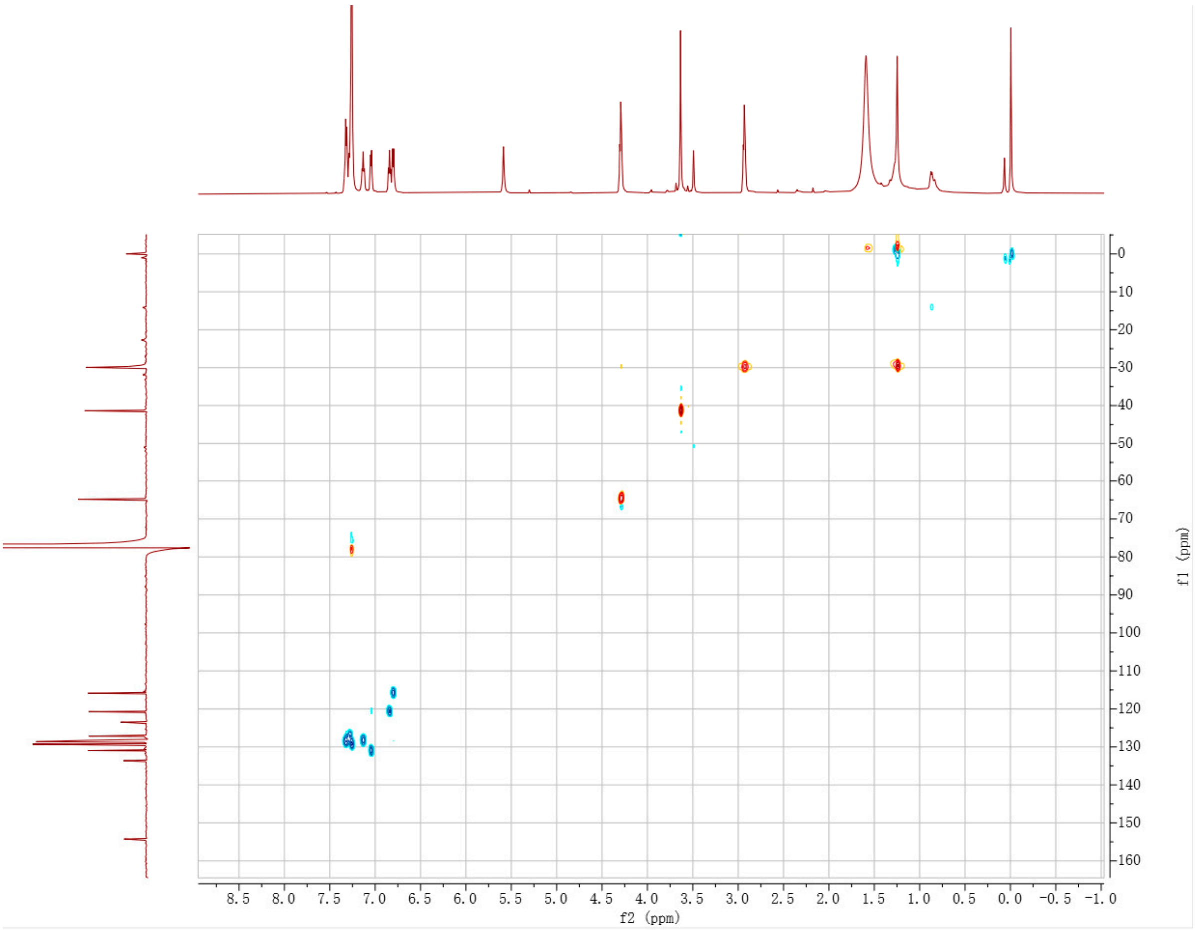

Figure S9 HSQC spectrum (600 MHz) of 2 in CDCl3.

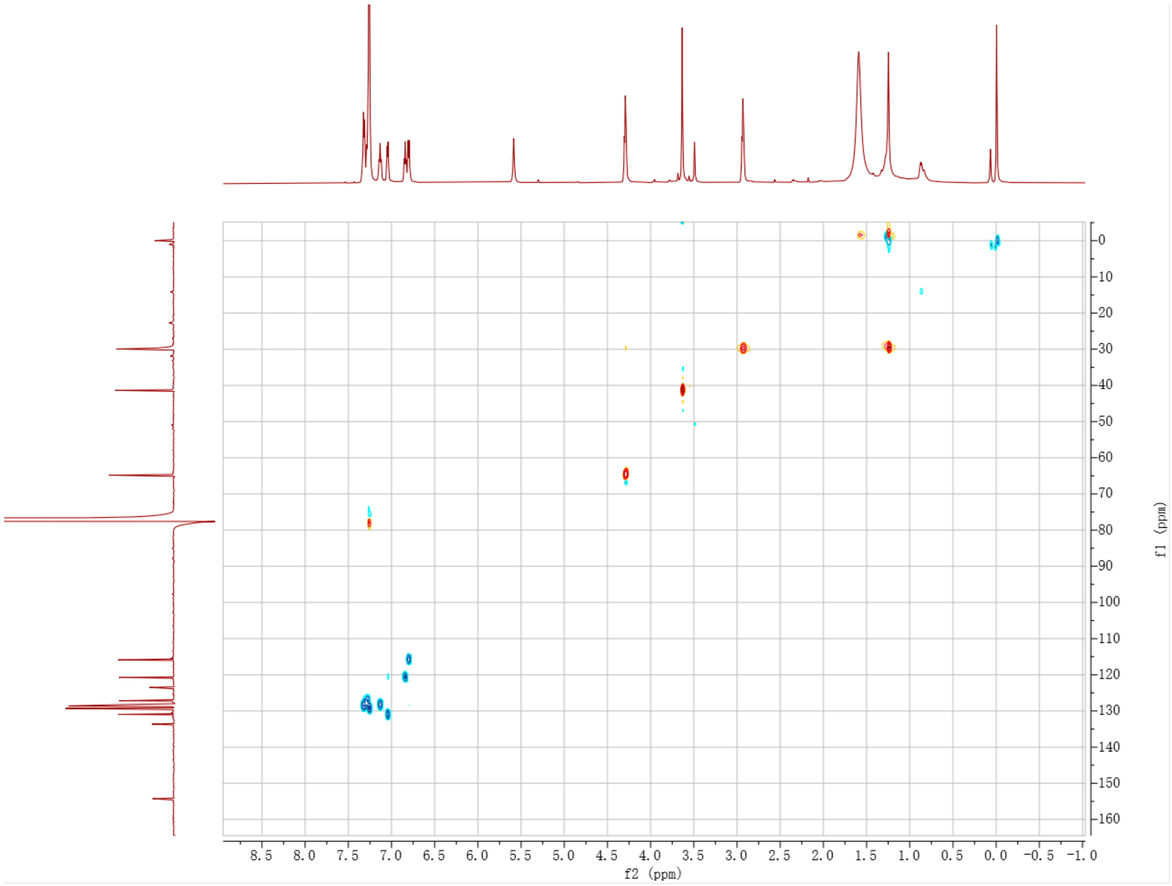

Figure S10 HMBC spectrum (600 MHz) of 2 in CDCl3.

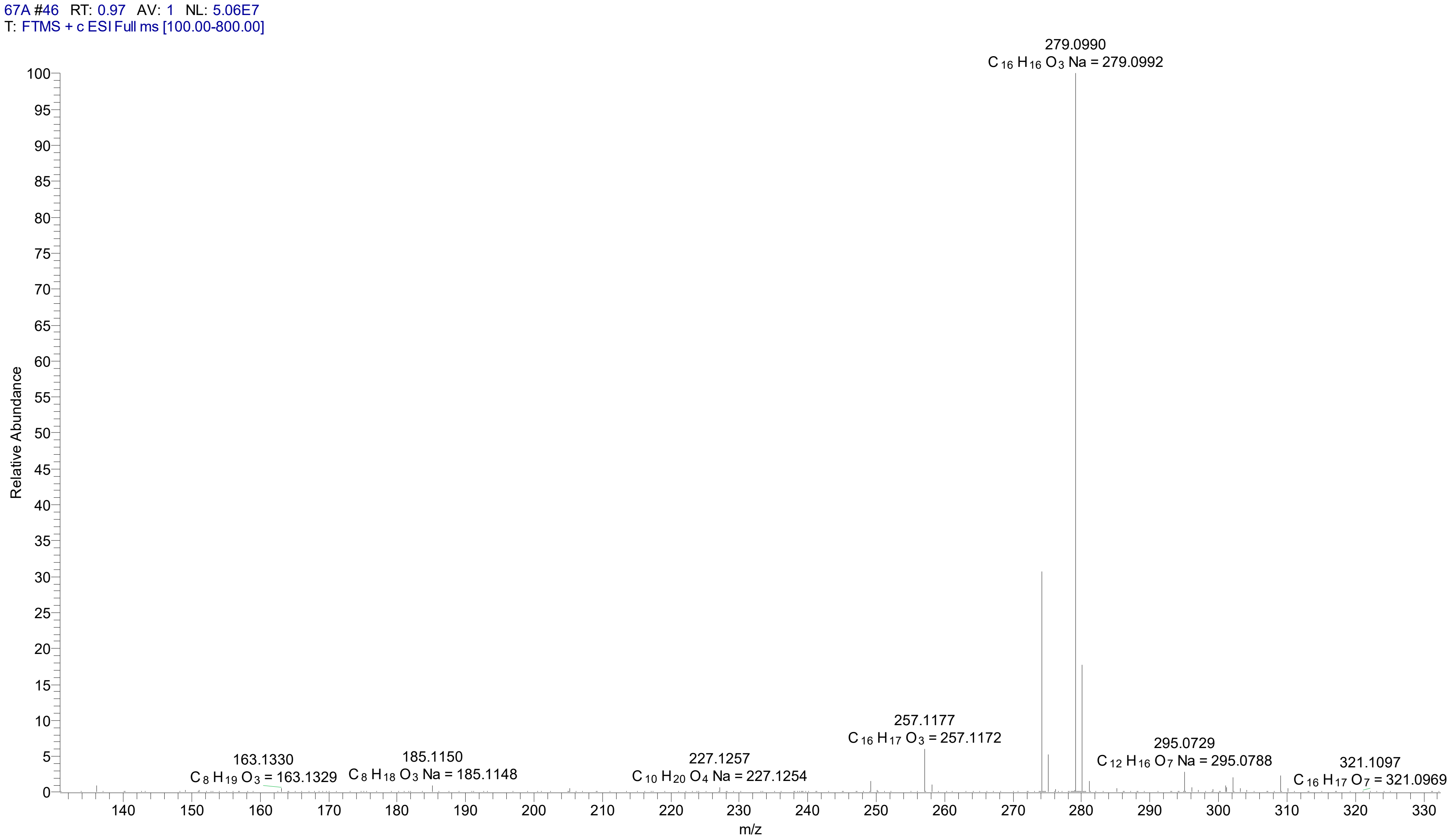

Figure S11 HRESIMS spectrum of 2.

6. NMR spectrum of compounds 3~24.

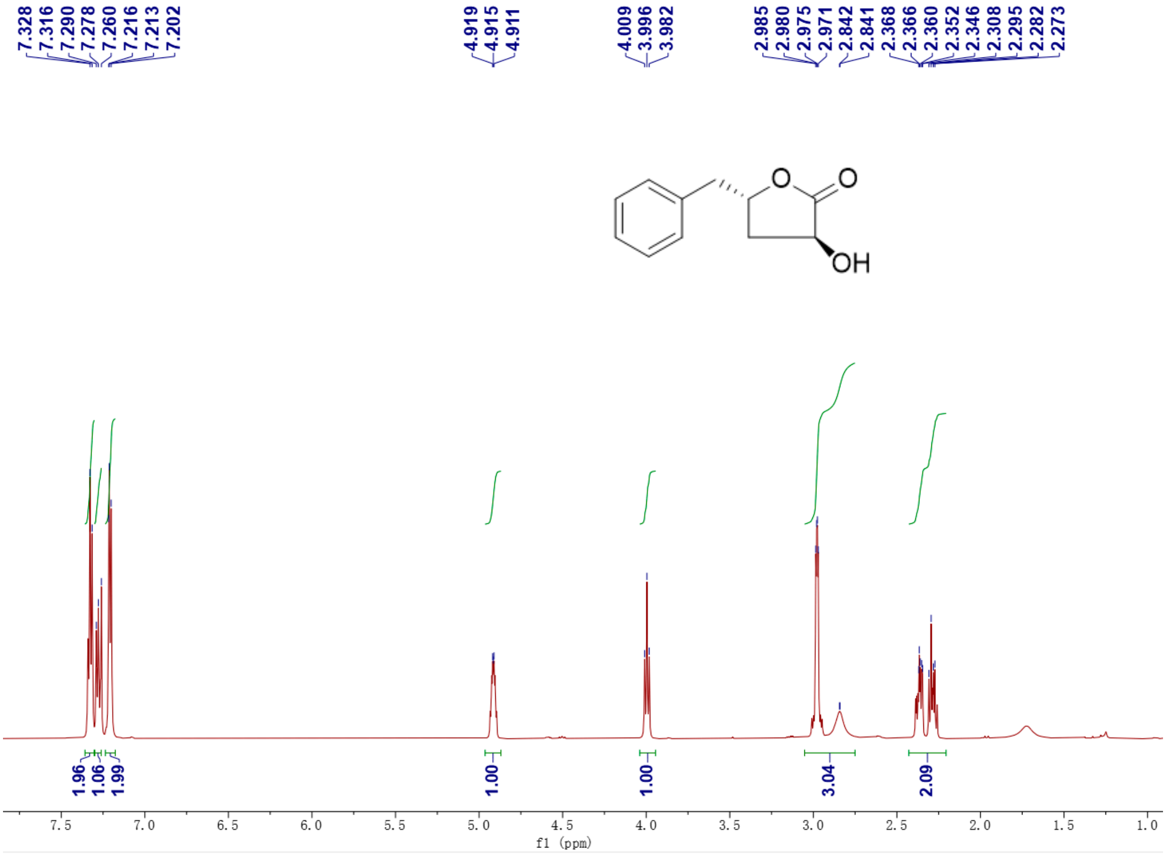

Figure S12 ^{1}H-NMR spectrum (500 MHz) of 3 in $CDCl_3$.

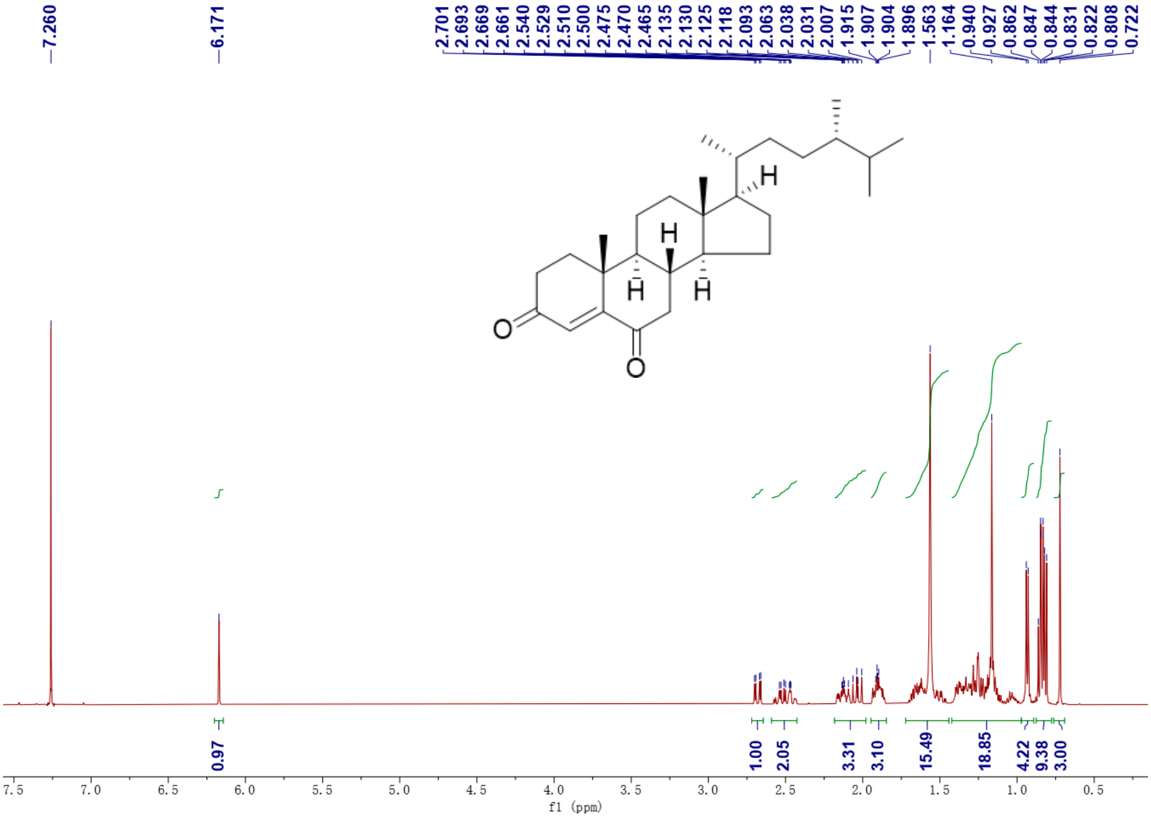

Figure S13 ^{1}H-NMR spectrum (500 MHz) of 4 in CDCl$_3$.

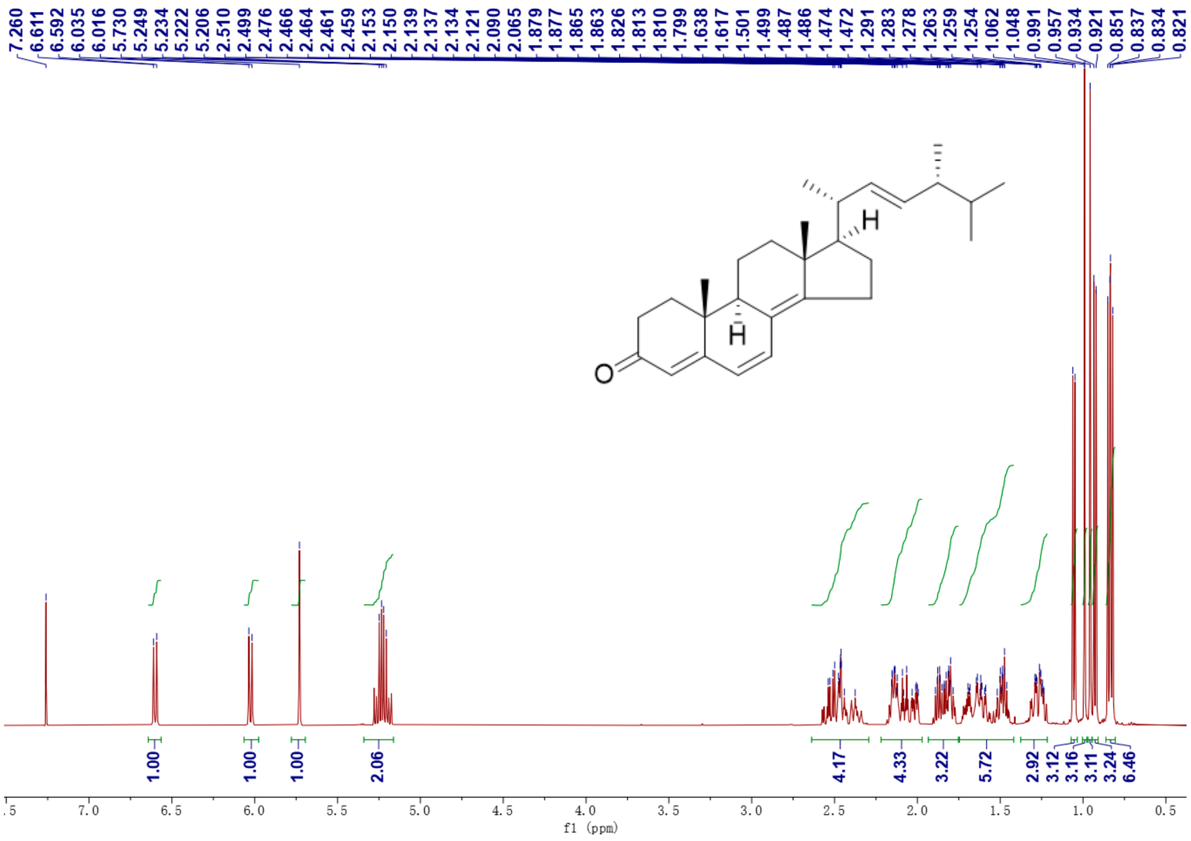

Figure S14 ^{1}H-NMR spectrum (500 MHz) of 5 in CDCl$_3$.

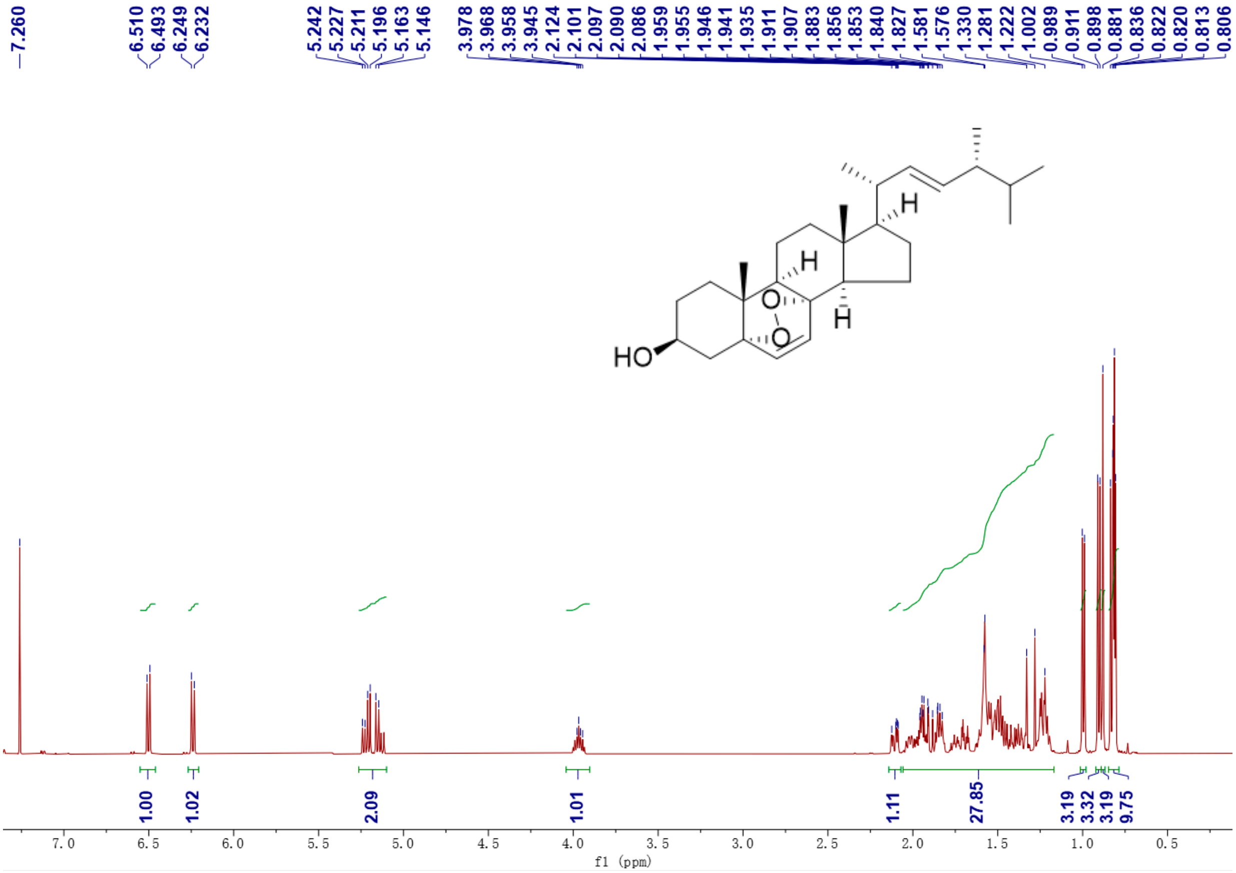

Figure S15 ^{1}H-NMR spectrum (500 MHz) of 6 in CDCl$_3$.

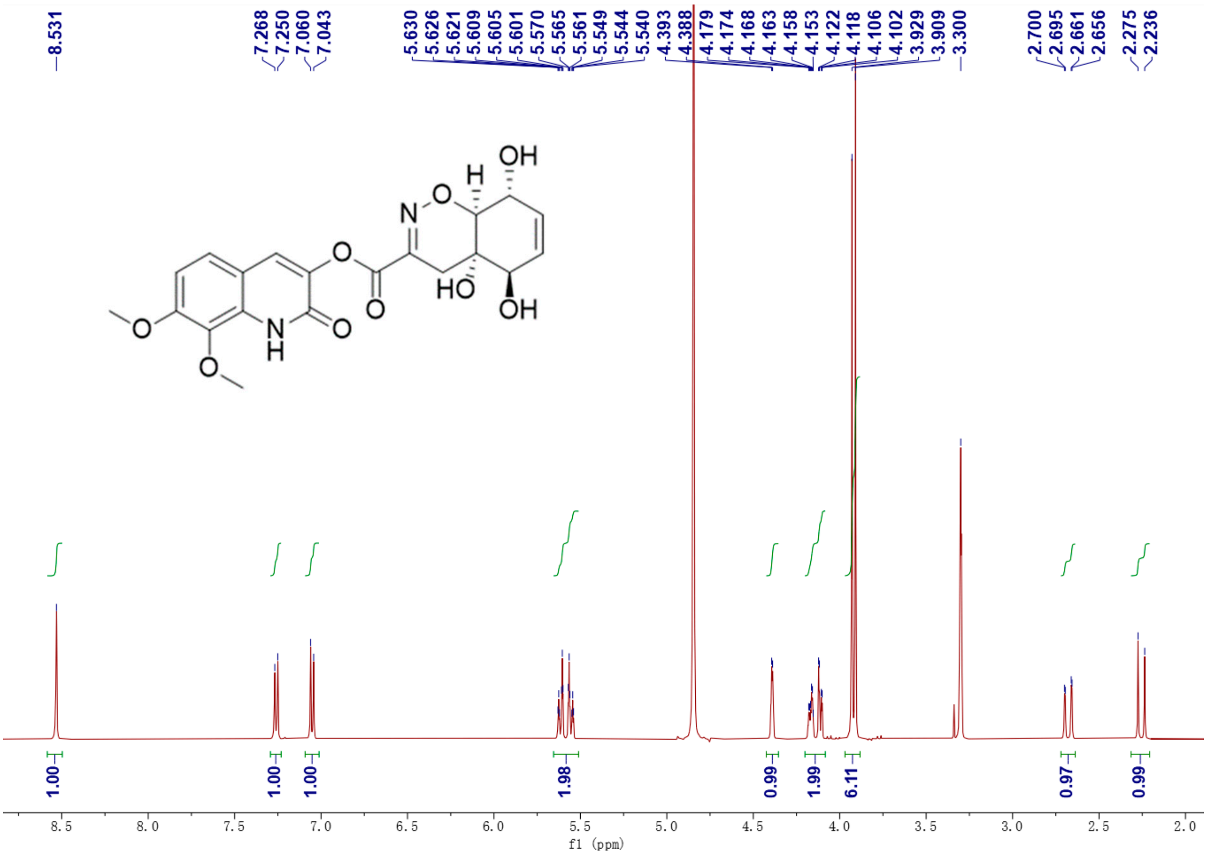

Figure S16 ¹H-NMR spectrum (500 MHz) of 7 in CD₃OD.

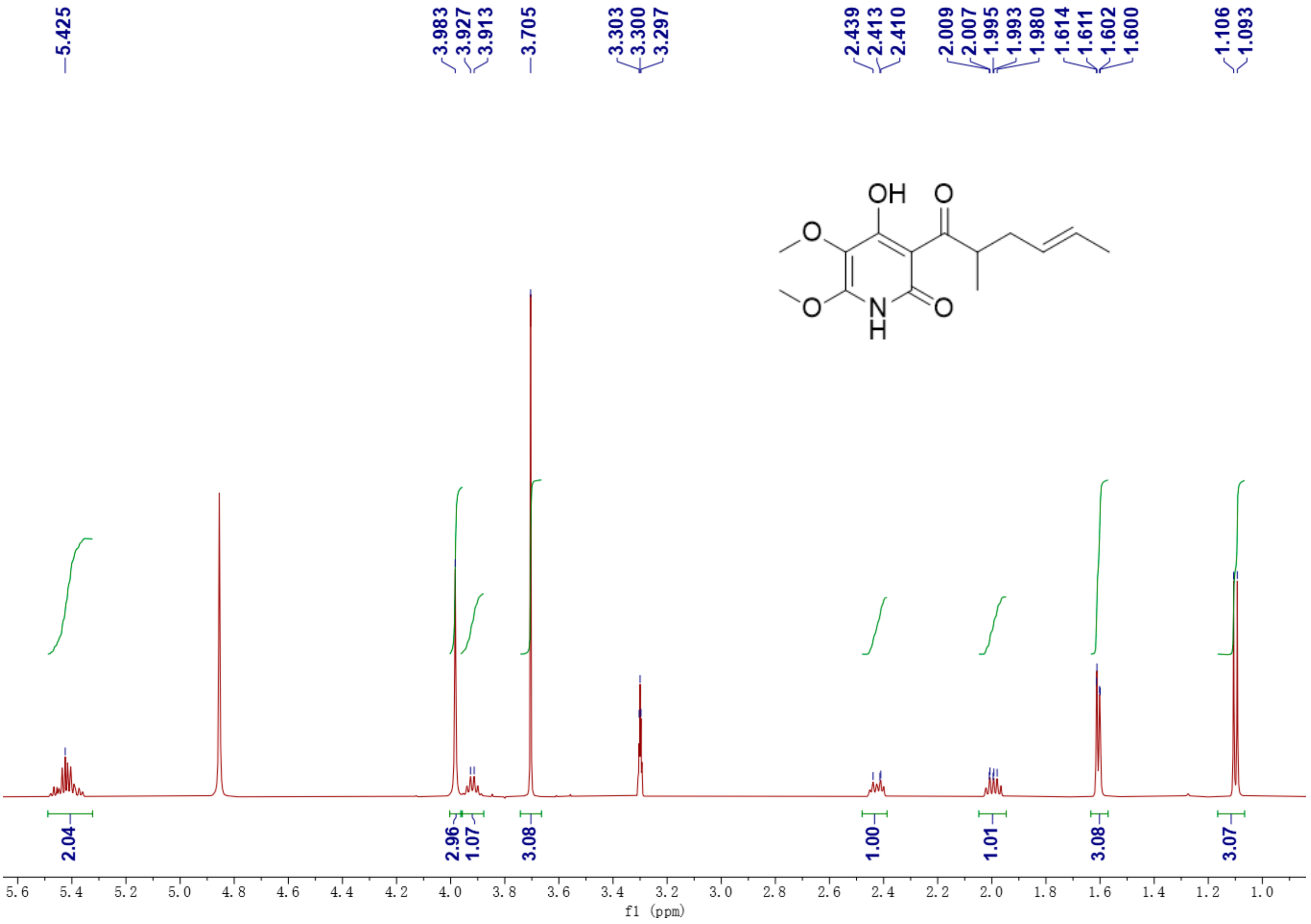

Figure S17 ¹H-NMR spectrum (500 MHz) of 8 in CD₃OD.

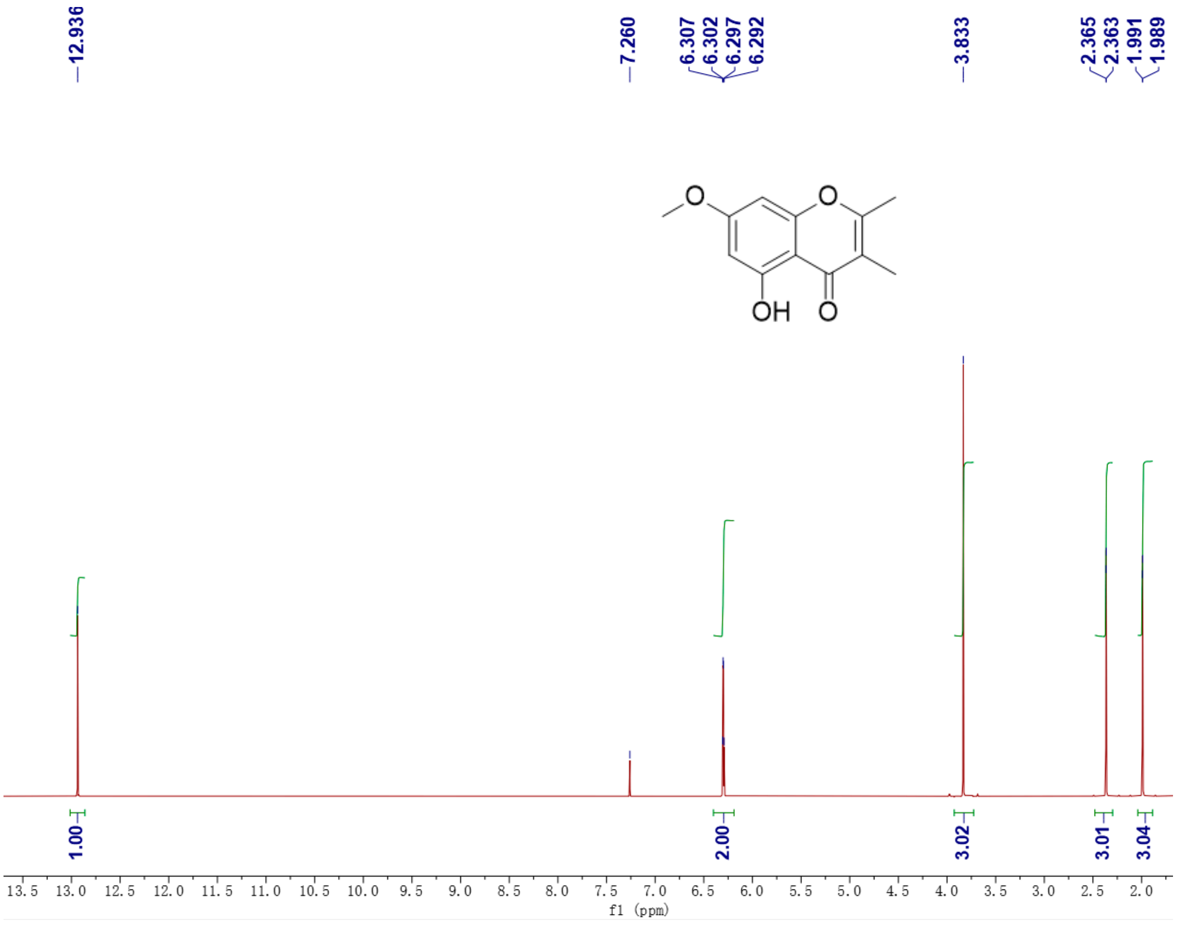

Figure S18 ^{1}H-NMR spectrum (500 MHz) of 9 in $CDCl_3$.

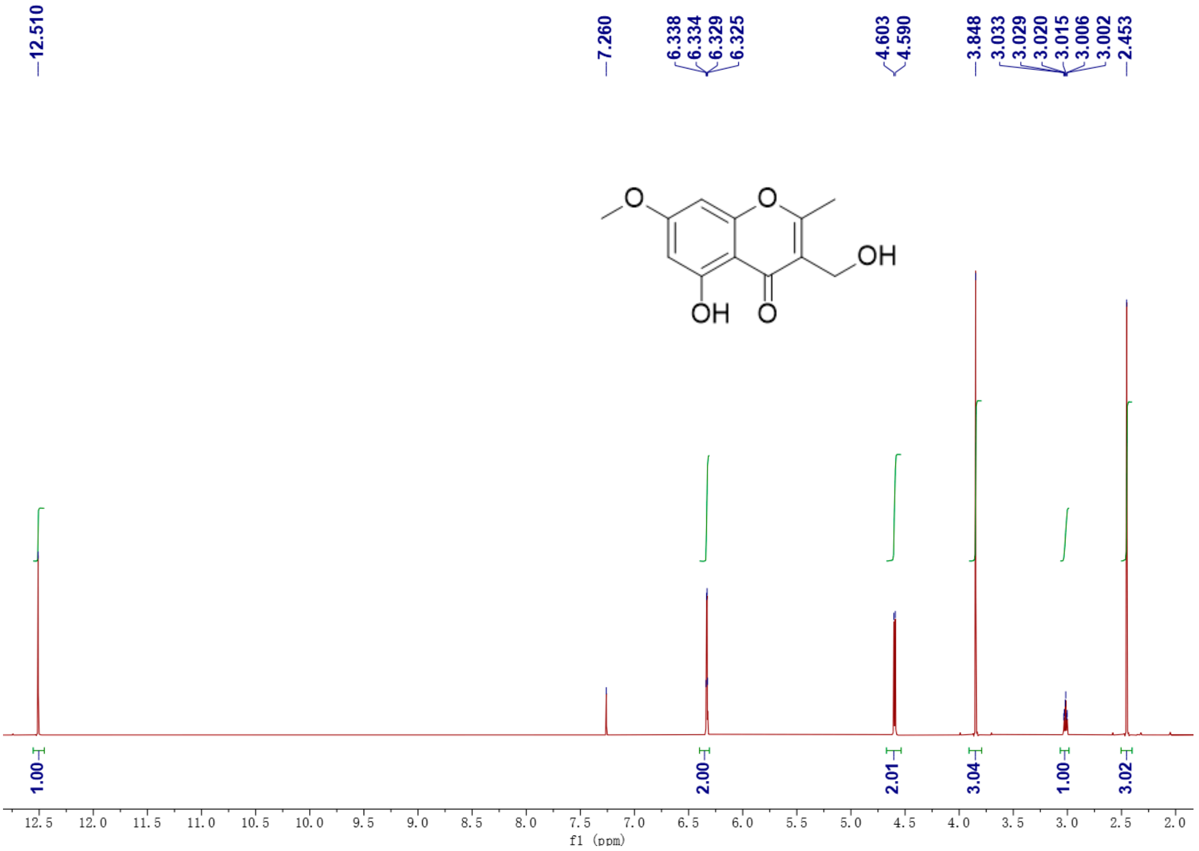

Figure S19 ^{1}H-NMR spectrum (500 MHz) of 10 in $CDCl_3$.

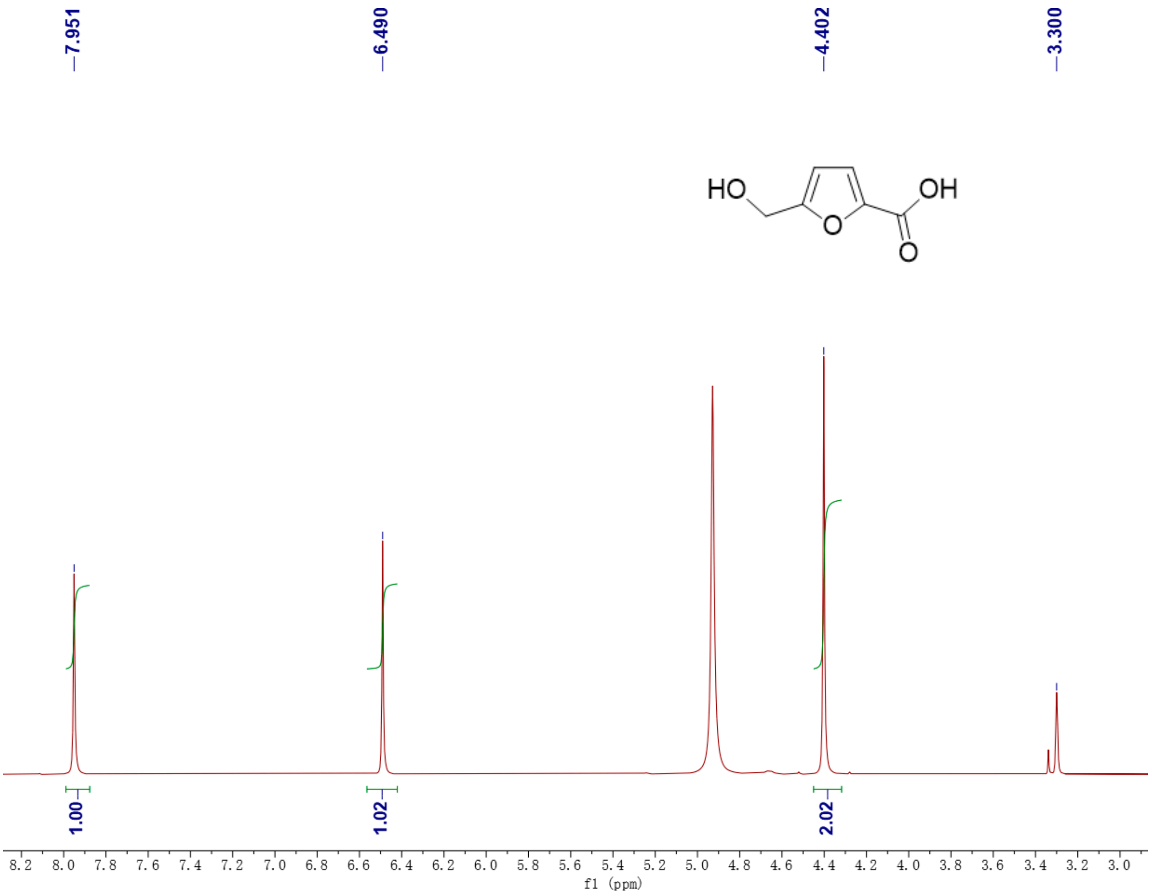

Figure S20 ^{1}H-NMR spectrum (500 MHz) of 11 in CD_3OD.

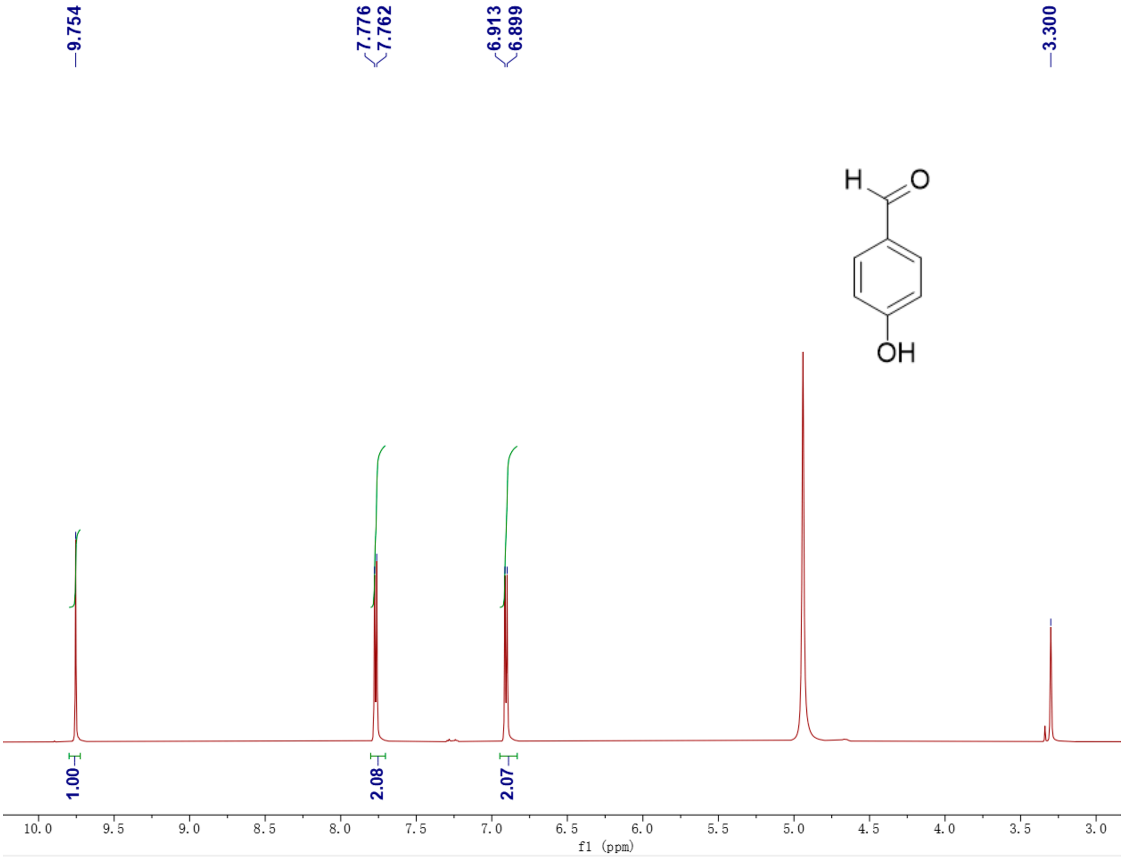

Figure S21 ^{1}H-NMR spectrum (500 MHz) of 12 in CD_3OD.

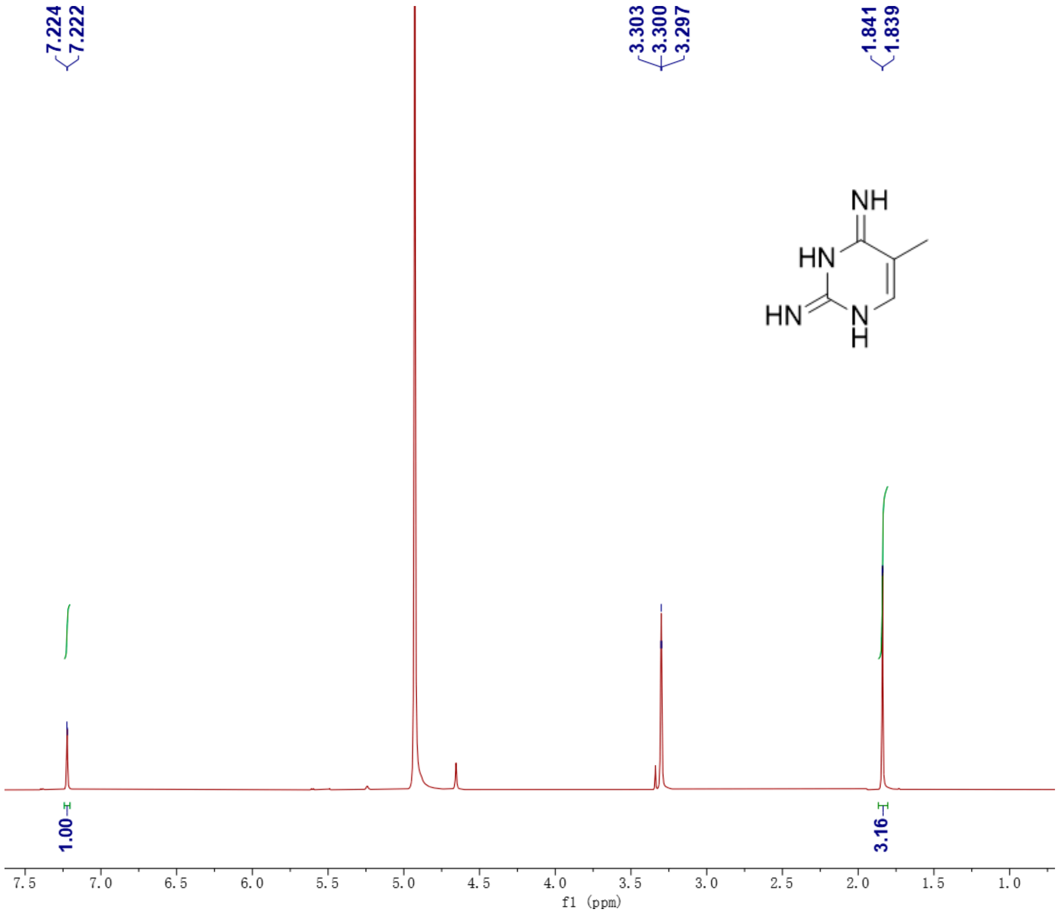

Figure S22 ^{1}H-NMR spectrum (500 MHz) of 13 in CD_3OD.

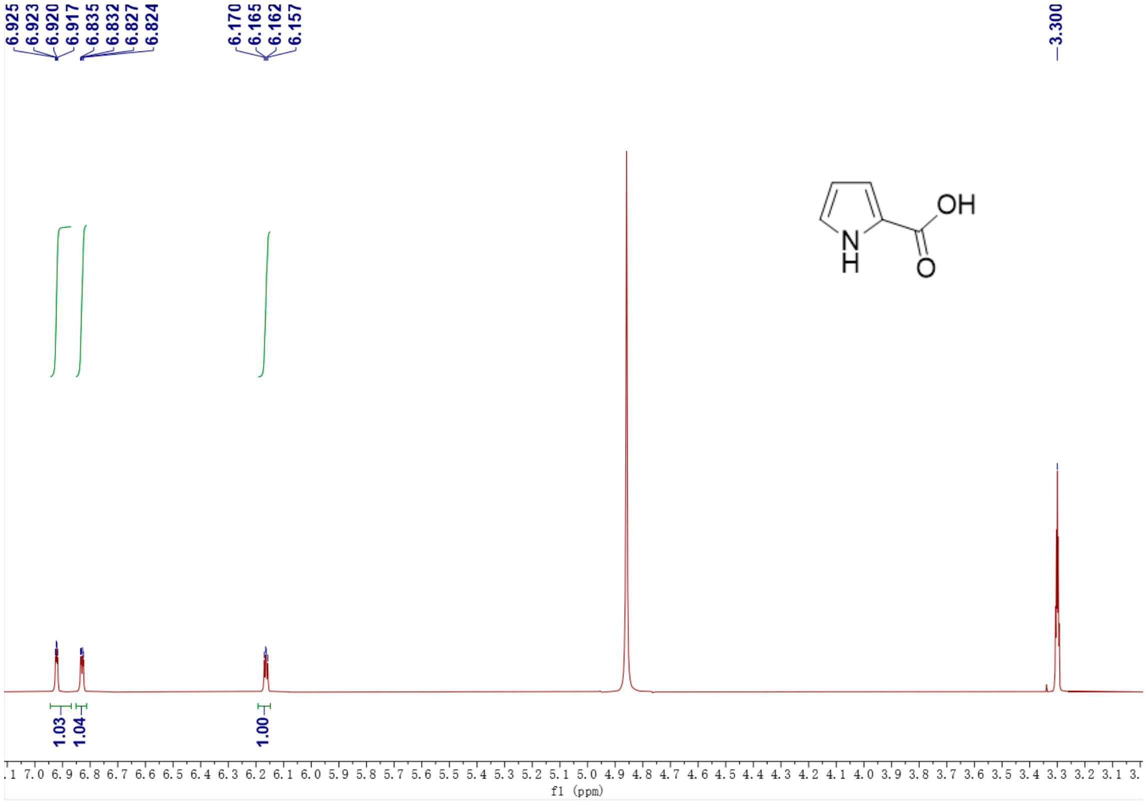

Figure S23 ^{1}H-NMR spectrum (500 MHz) of 14 in CD_3OD.

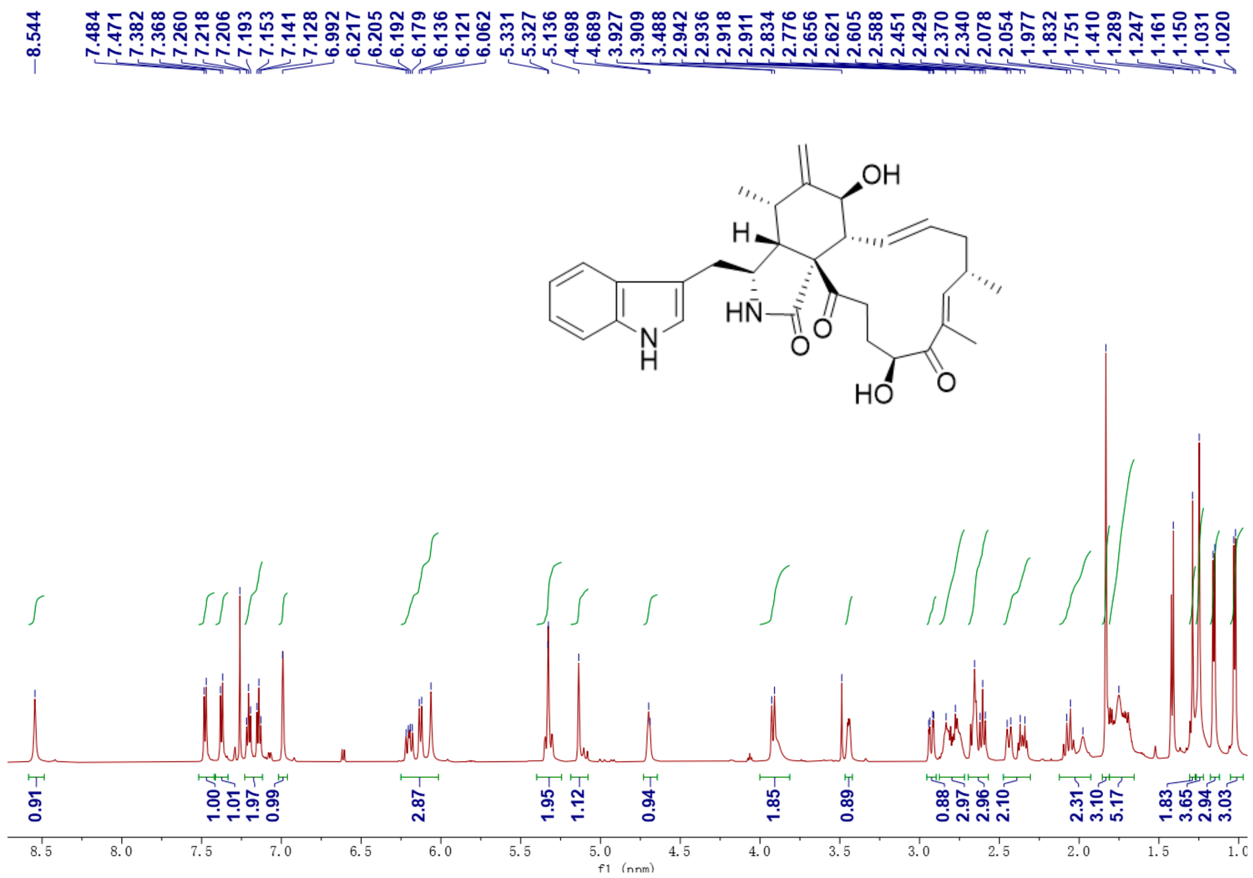

Figure S24 ^{1}H-NMR spectrum (500 MHz) of 15 in $CDCl_3$.

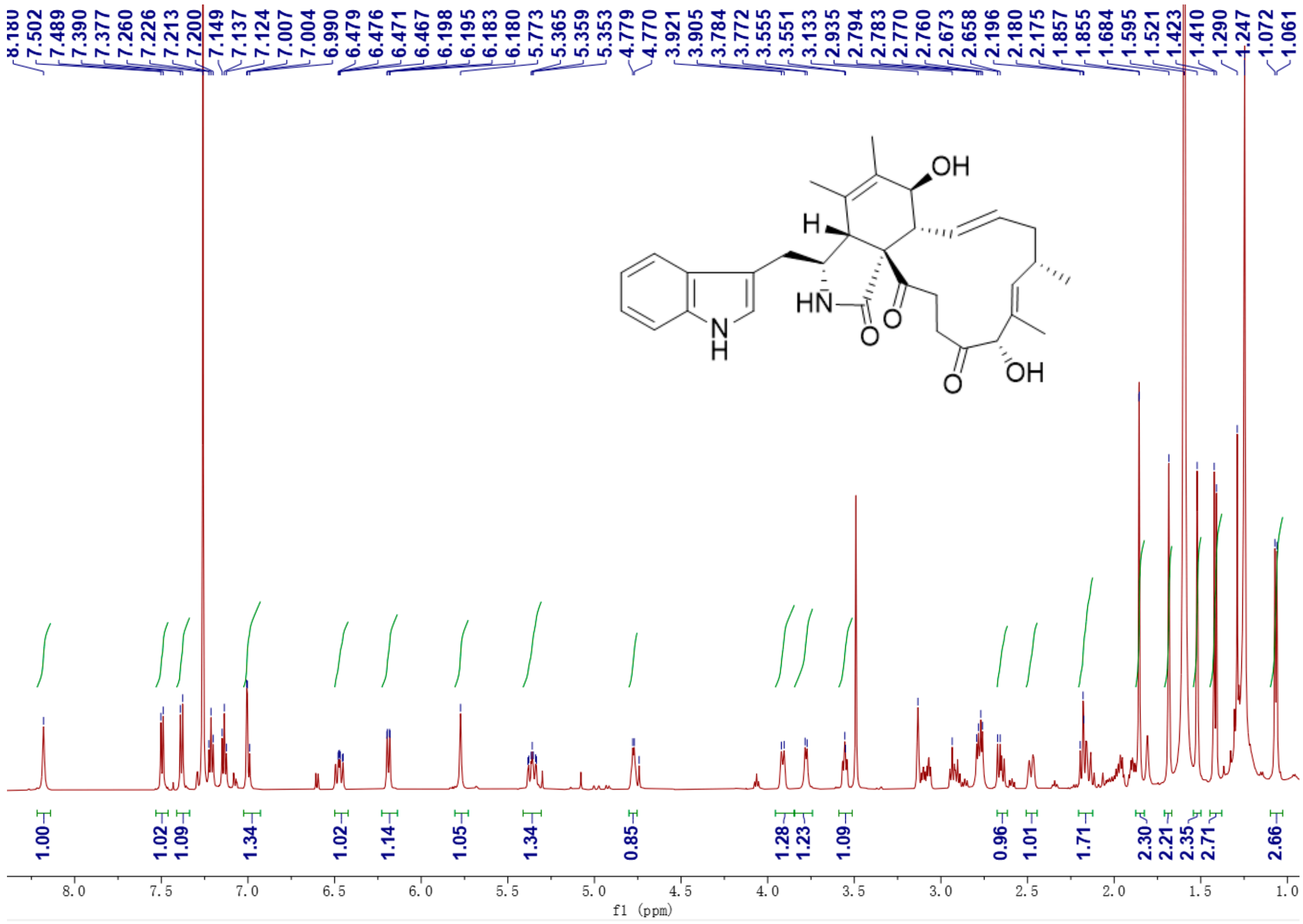

Figure S25 ^{1}H-NMR spectrum (500 MHz) of 16 in $CDCl_3$.

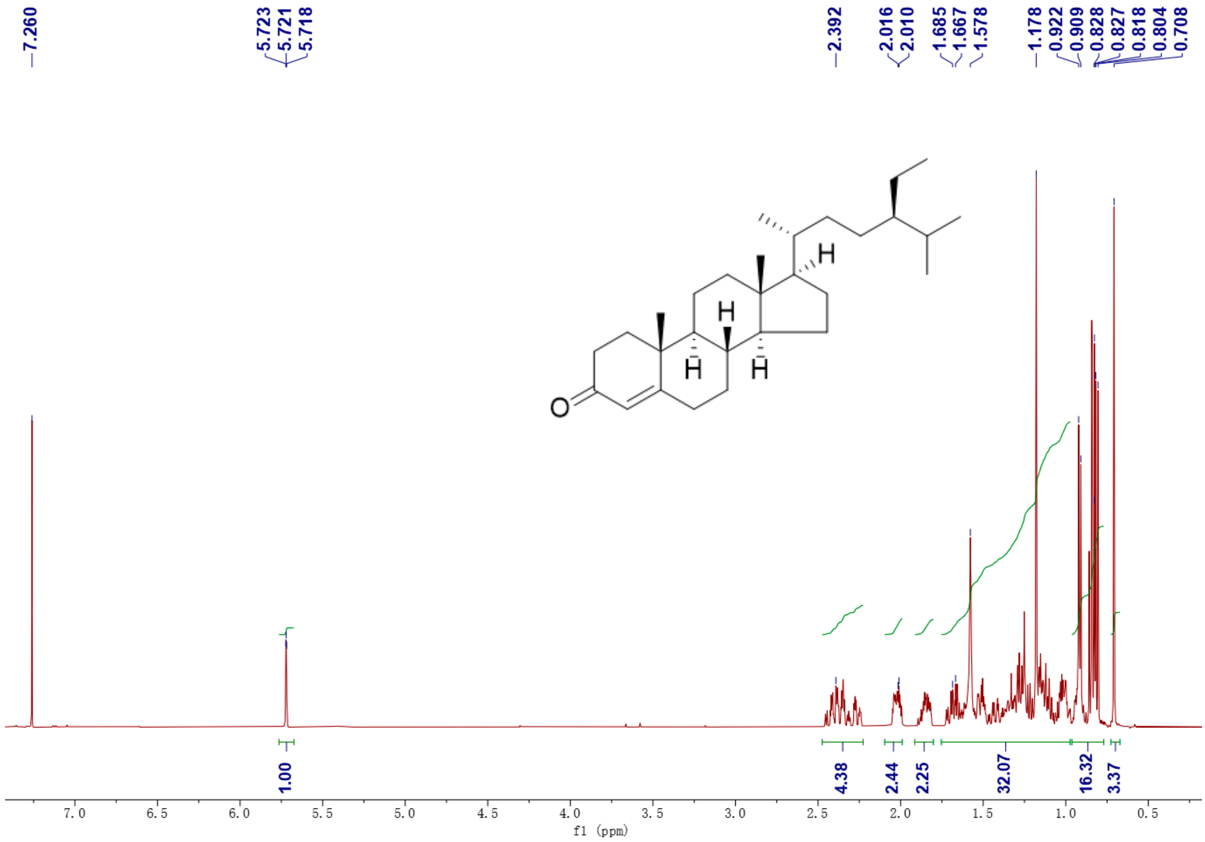

Figure S26 ^{1}H-NMR spectrum (500 MHz) of 17 in $CDCl_3$.

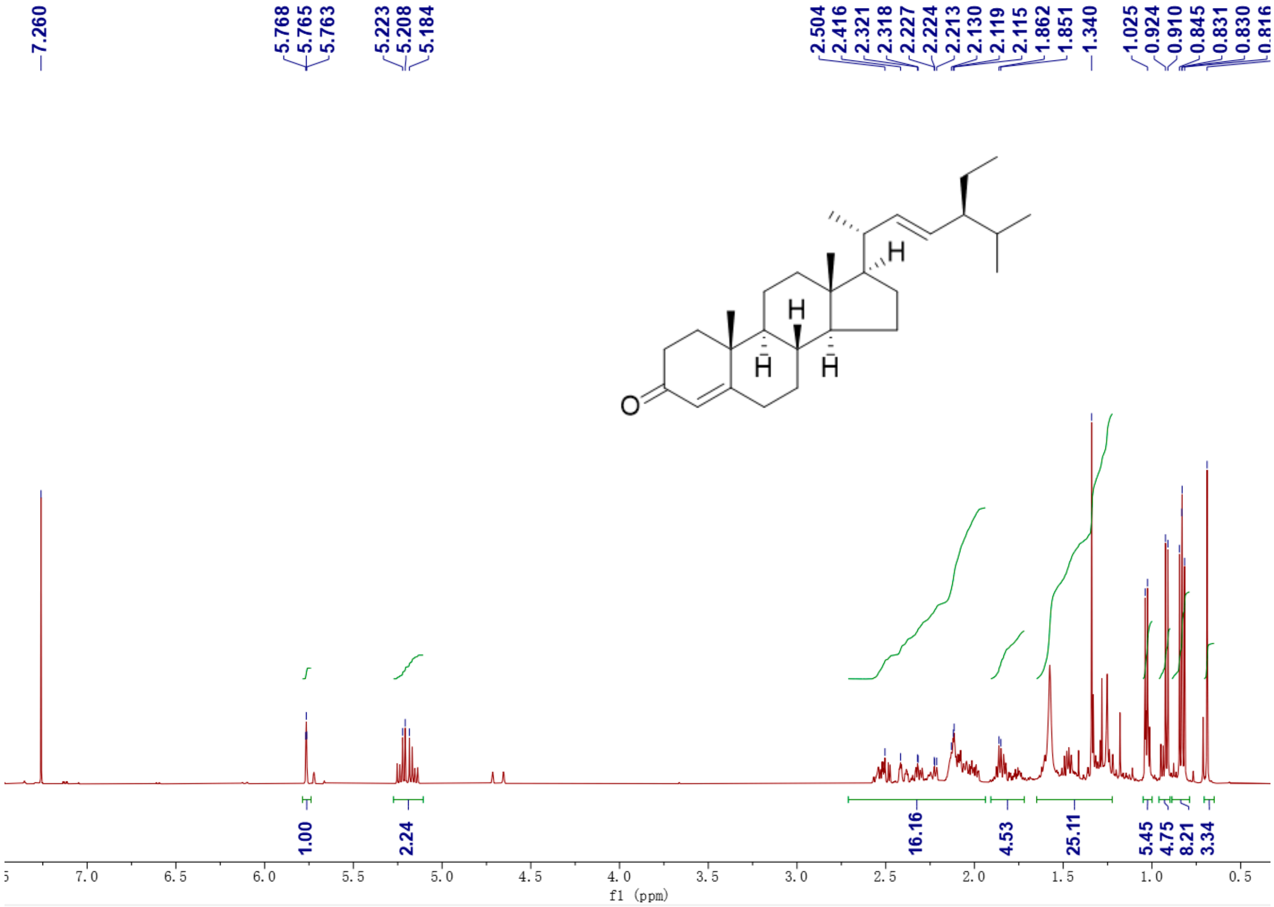

Figure S27 ^{1}H-NMR spectrum (500 MHz) of 18 in $CDCl_3$.

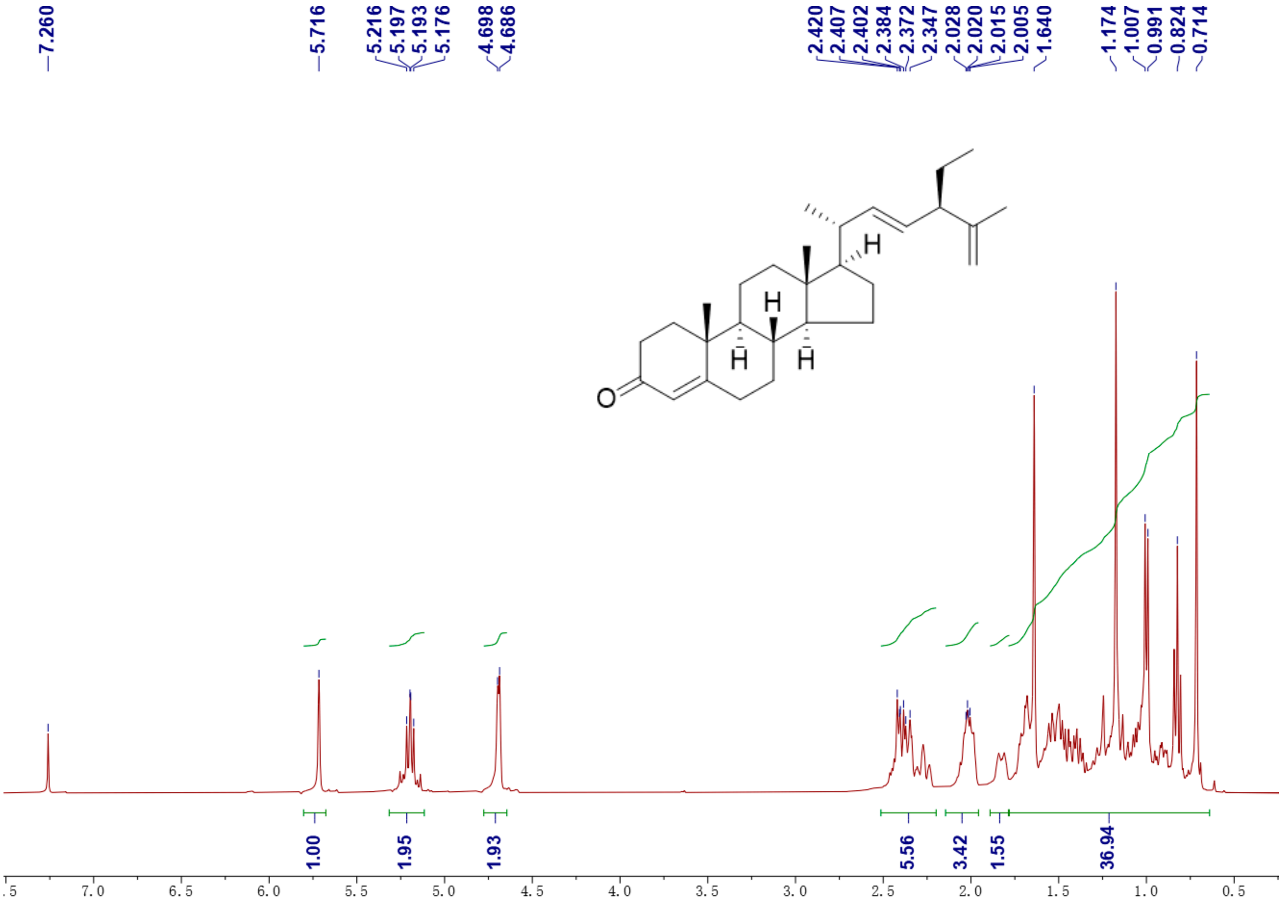

Figure S28 ¹H-NMR spectrum (500 MHz) of 19 in CDCl₃.

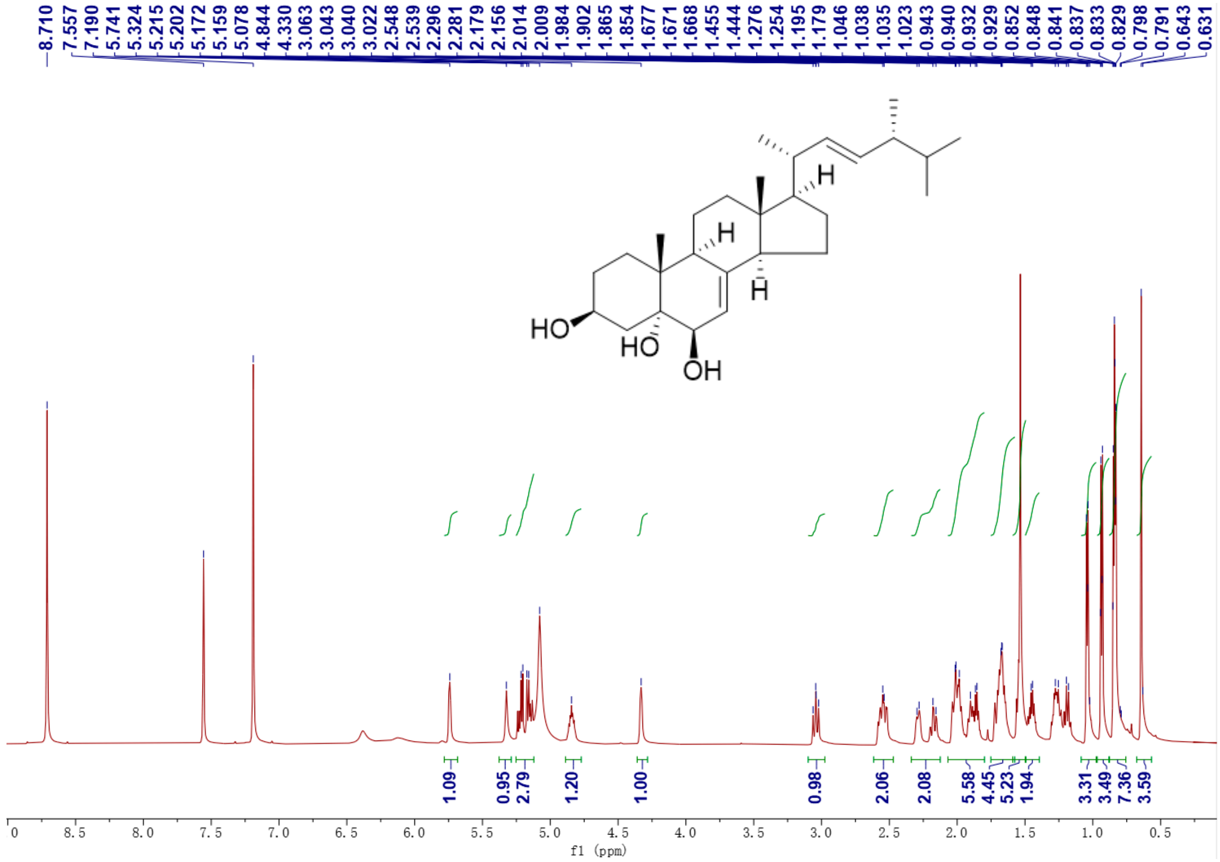

Figure S29 ¹H-NMR spectrum (500 MHz) of 20 in Pyridine-*d*₅.

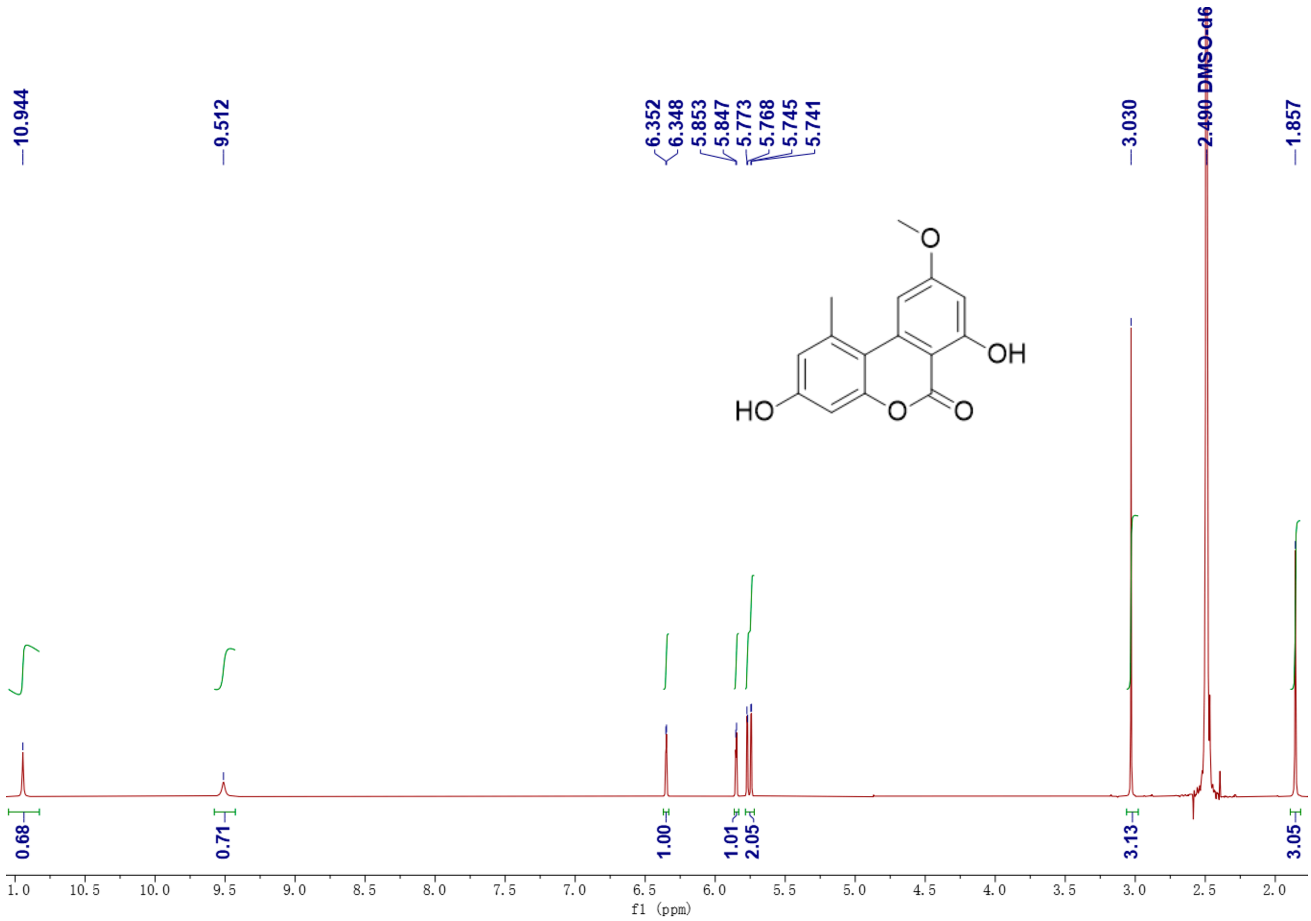

Figure S30 ¹H-NMR spectrum (500 MHz) of 21 in DMSO-d_6.

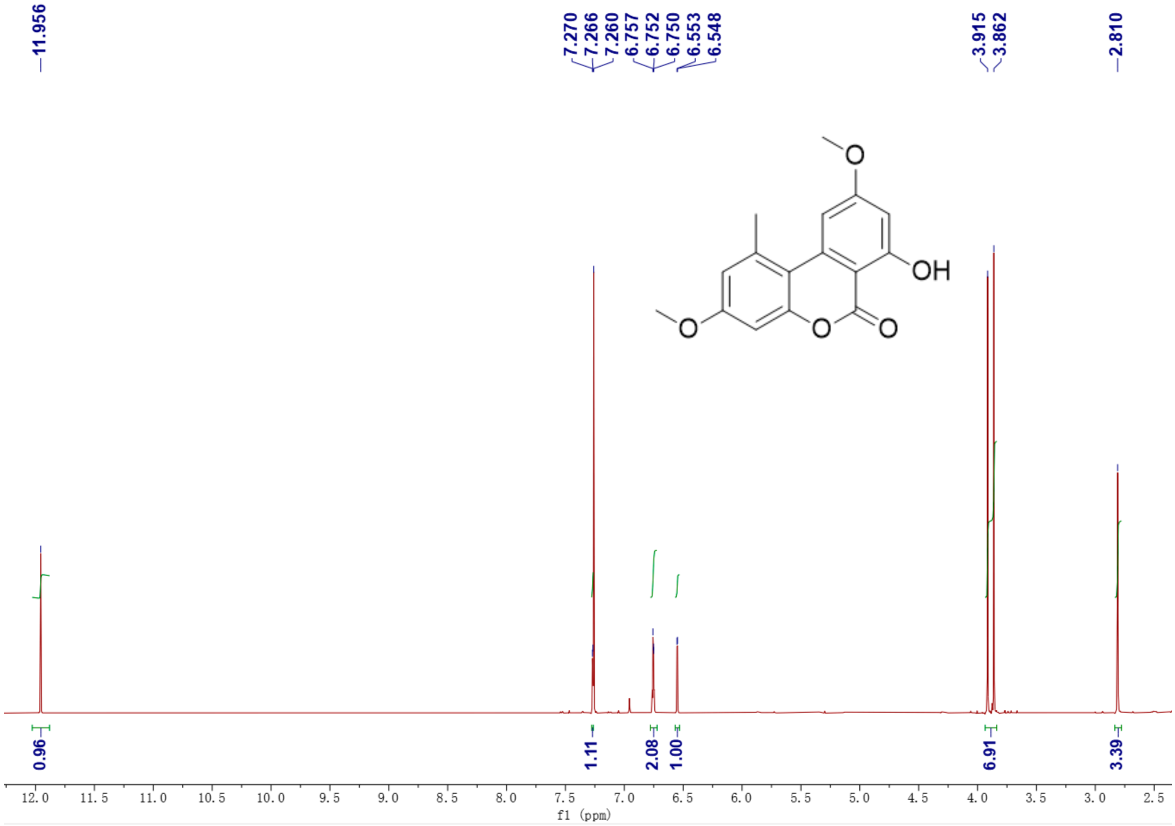

Figure S31 ¹H-NMR spectrum (500 MHz) of 22 in $CDCl_3$.

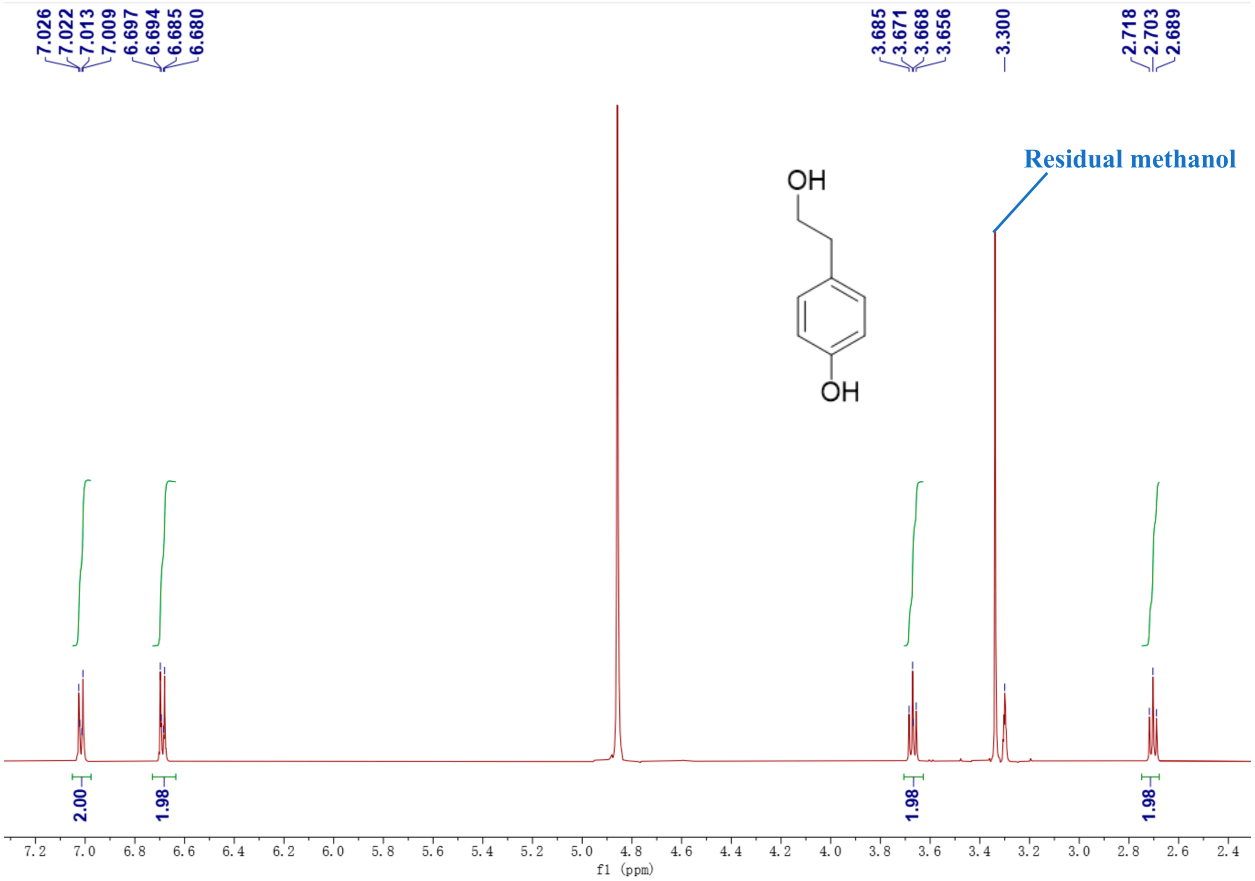

Figure S32 ^{1}H-NMR spectrum (500 MHz) of 23 in CD_3OD.

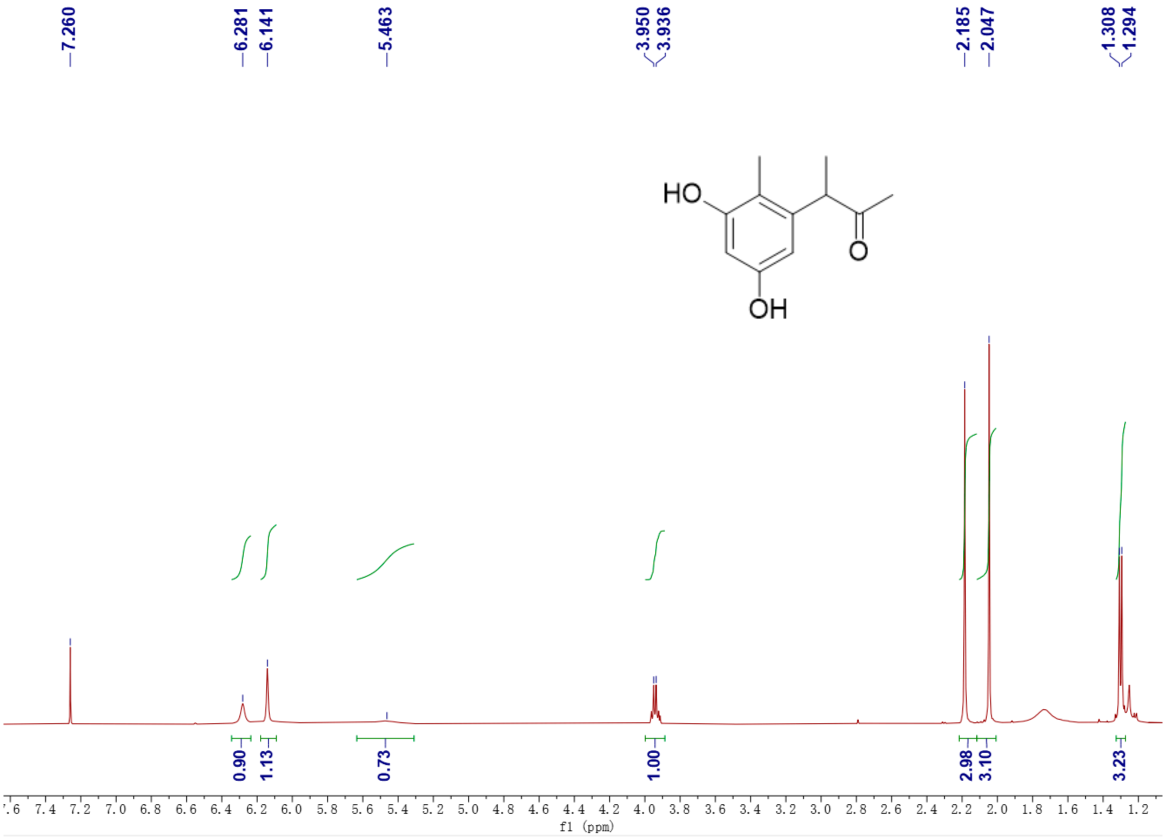

Figure S33 ^{1}H-NMR spectrum (500 MHz) of 24 in $CDCl_3$.

Figure S34 The gel electrophoresis of 16S rRNA gene amplicons

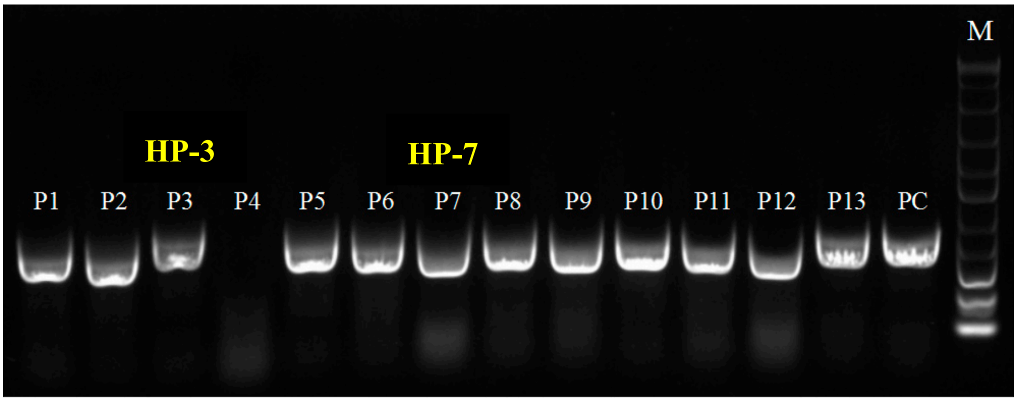

M: 250 bp DNA Ladder; PC: positive control

Figure S35 The sequence data analysis of HP-3

Trichoderma afroharzianum CBS 124620 ITS region; from TYPE material

Sequence ID: NR_137304.1 Length: 589 Number of Matches: 1

Range 1: 28 to 588 GenBank Graphics ▼ Next Match ▲ Previous Match

Score	Expect	Identities	Gaps	Strand
1003 bits(543)	0.0	558/564(99%)	6/564(1%)	Plus/Plus

```
Query  12   CTCCC-AACCC-ATGTG-ACGTTACCAAACTGTTGCCTCGGCGGGATCTCTGCCCCGGGT  68
Sbjct  28   CTCCCAAACCCAATGTGAACGTTACCAAACTGTTGCCTCGGCGGGATCTCTGCCCCGGGT  87

Query  69   GCGTCGCAGCCCCGGACCAAGGCGCCCGCCGGAGGACCAACCAAAACTCTTATTGTATAC  128
Sbjct  88   GCGTCGCAGCCCCGGACCAAGGCGCCCGCCGGAGGACCAACCAAAACTCTTATTGTATAC  147

Query  129  CCCCTCGCGGGttttttttATAATCTGAGCCTTCTCGGCGCCTCTCGTAGGCGTTTCGAA  188
Sbjct  148  CCCCTCGCGGG-TTTTTTTATAATCTGAGCCTTCTCGGCGCCTCTCGTAGGCGTTTCGAA  206

Query  189  AATGAATCAAAACTTTCAACAACGGATCTCTTGGTTCTGGCATCGATGAAGAACGCAGCG  248
Sbjct  207  AATGAATCAAAACTTTCAACAACGGATCTCTTGGTTCTGGCATCGATGAAGAACGCAGCG  266

Query  249  AAATGCGATAAGTAATGTGAATTGCAGAATTCAGTGAATCATCGAATCTTTGAACGCACA  308
Sbjct  267  AAATGCGATAAGTAATGTGAATTGCAGAATTCAGTGAATCATCGAATCTTTGAACGCACA  326

Query  309  TTGCGCCCGCCAGTATTCTGGCGGGCATGCCTGTCCGAGCGTCATTTCAACCCTCGAACC  368
Sbjct  327  TTGCGCCCGCCAGTATTCTGGCGGGCATGCCTGTCCGAGCGTCATTTCAACCCTCGAACC  386

Query  369  CCTCCGGGGGGTCGGCGTTGGGGATCGGCCCTGCCTCTTGGCGGTGGCCGTCTCCGAAAT  428
Sbjct  387  CCTCCGGGGGGTCGGCGTTGGGGATCGGCCCTGC--CTTGGCGGTGGCCGTCTCCGAAAT  444

Query  429  ACAGTGGCGGTCTCGCCGCAGCCTCTCCTGCGCAGTAGTTTGCACACTCGCATCGGGAGC  488
Sbjct  445  ACAGTGGCGGTCTCGCCGCAGCCTCTCCTGCGCAGTAGTTTGCACACTCGCATCGGGAGC  504

Query  489  GCGGCGCGTCCACAGCCGTTAAACACCCAACTTCTGAAATGTTGACCTCGGATCAGGTAG  548
Sbjct  505  GCGGCGCGTCCACAGCCGTTAAACACCCAACTTCTGAAATGTTGACCTCGGATCAGGTAG  564

Query  549  GAATACCCGCTGAACTTAAGCATA  572
Sbjct  565  GAATACCCGCTGAACTTAAGCATA  588
```

Figure S36 The sequence data analysis of HP-7

Alternaria alstroemeriae CBS 118809 ITS region; from TYPE material

Sequence ID: NR_163686.1 **Length:** 569 **Number of Matches:** 1

Range 1: 53 to 569 GenBank Graphics ▼ Next Match ▲ Previous Match

Score	Expect	Identities	Gaps	Strand
944 bits(511)	0.0	516/518(99%)	2/518(0%)	Plus/Plus

```
Query  13    AGGCGGGCTGG-ACCTCTCGGGGTTACAGCCTTGCTGAATTATTCACCCTTGTCTTTTGC  71
             ||||||||||| ||||||||||||||||||||||||||||||||||||||||||||||||
Sbjct  53    AGGCGGGCTGGAACCTCTCGGGGTTACAGCCTTGCTGAATTATTCACCCTTGTCTTTTGC  112

Query  72    GTACTTCTTGTTTCCTTGGTGGGTTCGCCCACCACTAGGACAAACATAAACCTTTTGTAA  131
             ||||||||||||||||||||||||||||||||||||||||||||||||||||||||||||
Sbjct  113   GTACTTCTTGTTTCCTTGGTGGGTTCGCCCACCACTAGGACAAACATAAACCTTTTGTAA  172

Query  132   TTGCAATCAGCGTCAGTAACAAATTAATAATTACAACTTTCAACAACGGATCTCTTGGTT  191
             ||||||||||||||||||||||||||||||||||||||||||||||||||||||||||||
Sbjct  173   TTGCAATCAGCGTCAGTAACAAATTAATAATTACAACTTTCAACAACGGATCTCTTGGTT  232

Query  192   CTGGCATCGATGAAGAACGCAGCGAAATGCGATAAGTAGTGTGAATTGCAGAATTCAGTG  251
             ||||||||||||||||||||||||||||||||||||||||||||||||||||||||||||
Sbjct  233   CTGGCATCGATGAAGAACGCAGCGAAATGCGATAAGTAGTGTGAATTGCAGAATTCAGTG  292

Query  252   AATCATCGAATCTTTGAACGCACATTGCGCCCTTTGGTATTCCAAAGGGCATGCCTGTTC  311
             ||||||||||||||||||||||||||||||||||||||||||||||||||||||||||||
Sbjct  293   AATCATCGAATCTTTGAACGCACATTGCGCCCTTTGGTATTCCAAAGGGCATGCCTGTTC  352

Query  312   GAGCGTCATTTGTACCCTCAAGCTTTGCTTGGTGTTGGGCGTCTTGTCTCTAGCTTTGCT  371
             ||||||||||||||||||||||||||||||||||||||||||||||||||||||||||||
Sbjct  353   GAGCGTCATTTGTACCCTCAAGCTTTGCTTGGTGTTGGGCGTCTTGTCTCTAGCTTTGCT  412

Query  372   GGAGACTCGCCTTAAAGTAATTGGCAGCCGGCCTACTGGTTTCGGAGCGCAGCACAAGTC  431
             ||||||||||||||||||||||||||||||||||||||||||||||||||||||||||||
Sbjct  413   GGAGACTCGCCTTAAAGTAATTGGCAGCCGGCCTACTGGTTTCGGAGCGCAGCACAAGTC  472

Query  432   GCACTCTCTATCAGCAAAGGTCTAGCATCCATTAAGCCtttttttCAACTTTTGACCTCG  491
             |||||||||||||||||||||||||||||||||||||| |||||||||||||||||||||
Sbjct  473   GCACTCTCTATCAGCAAAGGTCTAGCATCCATTAAGCC-TTTTTTCAACTTTTGACCTCG  531

Query  492   GATCAGGTAGGGATACCCGCTGAACTTAAGCATATCAA  529
             ||||||||||||||||||||||||||||||||||||||
Sbjct  532   GATCAGGTAGGGATACCCGCTGAACTTAAGCATATCAA  569
```
